# Association between major surgical admissions and the cognitive trajectory: 19 year follow-up of Whitehall II cohort study

**DOI:** 10.1136/bmj.l4466

**Published:** 2019-08-07

**Authors:** Bryan M Krause, Séverine Sabia, Helen J Manning, Archana Singh-Manoux, Robert D Sanders

**Affiliations:** 1 Department of Anesthesiology, University of Wisconsin, Madison, WI 53792-3272, USA; 2 Inserm U1153, Epidemiology of Ageing and Neurodegenerative diseases, Université de Paris, France; 3 Department of Epidemiology and Public Health, University College London, London, UK; 4 Department of Obstetrics and Gynecology, University of Wisconsin, Madison, WI, USA

## Abstract

**Objective:**

To quantify the association between major surgery and the age related cognitive trajectory.

**Design:**

Prospective longitudinal cohort study.

**Setting:**

United Kingdom.

**Participants:**

7532 adults with as many as five cognitive assessments between 1997 and 2016 in the Whitehall II study, with linkage to hospital episode statistics. Exposures of interest included any major hospital admission, defined as requiring more than one overnight stay during follow-up.

**Main outcomes measures:**

The primary outcome was the global cognitive score established from a battery of cognitive tests encompassing reasoning, memory, and phonemic and semantic fluency. Bayesian linear mixed effects models were used to calculate the change in the age related cognitive trajectory after hospital admission. The odds of substantial cognitive decline induced by surgery defined as more than 1.96 standard deviations from a predicted trajectory (based on the first three cognitive waves of data) was also calculated.

**Results:**

After accounting for the age related cognitive trajectory, major surgery was associated with a small additional cognitive decline, equivalent on average to less than five months of aging (95% credible interval 0.01 to 0.73 years). In comparison, admissions for medical conditions and stroke were associated with 1.4 (1.0 to 1.8) and 13 (9.6 to 16) years of aging, respectively. Substantial cognitive decline occurred in 2.5% of participants with no admissions, 5.5% of surgical admissions, and 12.7% of medical admissions. Compared with participants with no major hospital admissions, those with surgical or medical events were more likely to have substantial decline from their predicted trajectory (surgical admissions odds ratio 2.3, 95% credible interval 1.4 to 3.9; medical admissions 6.2, 3.4 to 11.0).

**Conclusions:**

Major surgery is associated with a small, long term change in the average cognitive trajectory that is less profound than for major medical admissions. The odds of substantial cognitive decline after surgery was about doubled, though lower than for medical admissions. During informed consent, this information should be weighed against the potential health benefits of surgery.

## Introduction

Cognitive decline and dementia are major healthcare concerns at older ages owing to considerable personal and societal burdens. Cognitive decline starts before conventional definitions of old age^1^ (often 65 years) and accelerates with aging and accumulation of comorbidities.^2^ ^3^ ^4^ Certain health events, such as stroke, can lead to profound changes in the cognitive trajectory such that there is a permanent “step change” in cognitive function.^5^ For 60 years a major concern has been that surgery might also drive long term changes in cognition^6^; our recent survey suggested that 65% of the public are concerned about postoperative cognitive deficits,^7^ perhaps leading to refusal of surgery that might otherwise have health benefits.^8^ ^9^ Yet studies investigating associations between surgery and long term cognitive outcomes have produced inconsistent results, with reports of cognitive harm,^10^ ^11^ no effect,^12^ ^13^ ^14^ and cognitive improvement.^15^ Despite inconclusive evidence, considerable concern remains about the potential for surgery to induce cognitive impairment.^7^ ^16^ Longer life expectancy implies an increasing number of surgical operations in older adults, hence a better understanding of the extent of any change in cognition after surgery is urgently required.

Research on postoperative cognitive decline has several limitations. First, most studies have a single preoperative assessment of cognitive function^8^ ^17^ and not a person specific cognitive trajectory before the surgical event. Consequently, any decline detected postoperatively could be falsely attributed to the surgery rather than to the individual’s preoperative cognitive trajectory. Second, studies also typically fail to consider the impact of medical events^5^ ^18^ such as stroke,^5^ which likely have a large cognitive impact and cluster with major surgical events. Third, most studies are small, with a limited set of confounding factors. Fourth, studies often do not include “positive controls” to aid in the interpretation of any null or borderline finding. Finally, despite notable exceptions,^13^ ^14^ ^19^ the duration of cognitive follow-up is typically less than one year, limiting inference on the long term impact of surgery on cognition.

We addressed these concerns using cognitive data from 7532 adults, investigating whether incident major surgical admissions are related to long term changes in the cognitive trajectory, using five waves of cognitive assessments spanning approximately 20 years, with adjustment for major medical admissions. To facilitate interpretation of results, we translate effect estimates to equivalent years of cognitive aging and relate changes to the effect of stroke, an event with an established impact on cognition. Reference points such as these enable discussions of informed consent with patients, allowing them to weigh the risks of cognitive injury more easily. We primarily aimed to establish the mean population effect of major surgery on cognitive decline. As a secondary outcome we developed a binary outcome of substantial cognitive decline, more analogous methodologically to prior studies of postoperative cognitive decline^10^ and consistent with clinically important deviations from the age related cognitive trajectory.^20^ It allows some further correspondence to the prior literature and emphasizes cognitive changes that could impact quality of life.

## Methods

### Study design and participants

The Whitehall II study is a prospective cohort study comprised of employees from the British civil service in London based offices. A total of 10 308 people (6895 men and 3413 women, aged 35-55 at enrollment) were recruited between 1985 and 1988. In 1997, when participants were 45-69 years old, a cognitive test battery was introduced to the study and the test repeated four times. Age span across the follow-up was 44-86 years (median age 64); mean 3.8 assessments per person, maximum follow-up 19.4 years (mean 12.9 years).

### Exposures

The events (major surgical or admissions for medical conditions) were defined as hospital admissions requiring at least two overnight stays (excluding ambulatory or outpatient events) as identified in the hospital episode statistics database of National Health Service hospitals, which covers admissions in England, Scotland, and Wales. High quality data have been available since 1997, with audits of discharge reports indicating a 96% accuracy over our study period.^21^

Surgical admissions in hospital episode statistics were defined by Office of Population, Censuses, and Services (OPCS) codes (see appendix 1). Our primary definition of major surgery required a hospital admission of at least two nights (this being consistent with definitions currently used in major perioperative clinical trials^22^ ^23^ ^24^) linked to an OPCS code. Emergency admissions were identified by specific OPCS codes designated as an emergency procedure. Minor surgery was an OPCS coded admission that did not incur a minimum stay of two nights. Medical admissions were identified by ICD-10 codes (international classification of diseases, 10th revision) and similarly required a hospital admission of at least two nights.

To limit effects of transfers within hospitals, we linked any admissions within 14 days. If OPCS codes were identified during this time, we treated the entire admission as a surgical admission. This design was used to ensure that complications of surgical admissions were grouped with the operations but might, if anything, weight the analysis toward finding an exaggerated relation with surgery. We retained those admitted during the study period but without cognitive follow-up for use in adjusting baseline cognitive scores (such that surgical admissions thoughout the study period were treated the same when modeling cognitive scores, even if cognitive data were missing later).

We also conducted a sensitivity analysis rating procedures for surgical risk based on BUPA (a private health insurance scheme) scores as used in the surgical risk scale.^25^ Procedures were rated by two authors (RDS and HJM). The surgical risk scale includes various parameters to estimate, including patient comorbidities and the planned procedure, rated for severity on the BUPA scale. We have previously shown that higher BUPA rating of procedure severity (rated by RDS and HJM) is associated with higher risk of 30 day postoperative mortality.^26^ In our sensitivity analysis, the definition of major surgery required both a BUPA definition of a major procedure and a hospital admission of at least two nights. This analysis was designated as BUPA major.

Out of 43 692 entries in hospital episode statistics during the study period (for 7532 participants), 35 099 remained after linkage. We compared major surgical events for the entire group with those for whom cognitive follow-up was available, organized according to higher risk surgical categories that have plausible associations with cognitive outcomes (cardiac, thoracic, vascular, and intracranial operations).^10^ ^11^ The proportions were similar, suggesting that cognitive follow-up was available for a representative cross section of operations in the study population.

### Outcomes

The primary outcome was the global cognitive score, calculated from the cognitive domains tested (memory, executive function, and verbal fluency), as in previous analyses of these data.^1^ ^27^ ^28^ The cognitive test battery was administered in 1997-99 (age range 44-68 years), 2002-04, 2007-09, 2012-13, and 2015-16 (age range 62-86 years). In 1997-99, 556 participants underwent retesting within three months of their initial assessment, with good test-retest reliability (range 0.6-0.9). Memory was tested using a 20 word free recall test where one or two syllable words were presented at two second intervals and participants were asked to write down as many of the words they could recall in two minutes. Executive function was assessed using the Alice Heim 4-I test, which includes 65 verbal and mathematical reasoning items with increasing difficulty. This test measures a participant’s ability to identify patterns and infer principles or rules over the 10 minute test. Verbal fluency was assessed using measures of phonemic and semantic fluency, with participants asked to write as many words as possible beginning with “s” (phonemic) or animal names (semantic) in one minute. The primary outcome of the global cognitive score was calculated by first standardizing the raw scores for each cognitive domain to z scores using mean and standard deviation from the first wave of cognitive data collection (1997-99). Then we summed the z scores across cognitive domains and standardized them to yield the global score. This approach minimizes potential measurement error in any individual test.

### Covariates

Most covariates were drawn from the 1997-99 assessment, though we coded covariates such as diabetes as occurring “ever” based on all assessments. These comprised of sex, ethnicity, education level, maximum occupational position, diabetes mellitus, and smoking status. Additionally, measures of married or cohabitating status and Framingham cardiovascular disease risk score^29^ were updated alongside the cognitive assessment. We also included the number of cognitive assessments for each participant as a covariate.

### Statistical models

We estimated the offset in age related cognitive trajectory associated with cumulative major surgical, non-surgical, and stroke related hospital admissions. We report the 95% credible intervals on these estimates, which are close to confidence intervals derived using maximum likelihood or restricted maximum likelihood estimation. We used Markov chain Monte Carlo simulations to fit linear mixed effects models with random intercepts for participant and random slopes for age, accounting for variation between participants in baseline cognitive performance and in cognitive trajectory. Fixed effects represented the age related cognitive decline and the shift in cognitive performance according to the cumulative incidence of surgical and medical hospital admissions before the time of cognitive assessment. We separated medical admissions for stroke events because of the expected substantial cognitive impact after stroke.^5^ A quadratic term for age was included to account for an accelerated rate of decline with increasing age.^28^ Because our focus was on changes after surgery, we also included baseline adjustments for numbers of surgery, medical, and stroke admissions (including events that occurred after cognitive follow-up but during the range of years analyzed), and for two way interactions between them to ensure that the association observed is not attributable to differences by subgroups in preoperative cognitive function.

The form of the linear model is:

Cognition_ij_=β×[1+Age_ij_+Age_ij_^2^+(EverSurgery_i_+EverMedical_i_+EverStroke_i_)^2^+CognitiveAssessments_i_+(Surgery_ij_+Medical_ij_+Stroke_ij_)^2^+Covariates_i_+Covariates_i_×Age_ij_+CovariatesTD_ij_]+γ_i_×[1+Age_ij_]+ε_ij_

Grouped squared terms indicate two way interactions. Subscript i indicates predictors that vary across participants and subscript j indicates predictors that vary across cognitive assessments within one participant. “Ever” surgical admissions, medical events, and strokes represent occurrence of at least one of that event any time during the study period; therefore, these represent constant adjustments to the participant’s baseline. Covariates_i_ indicates adjustment for covariates that were measured at baseline, and CovariatesTD_ij_ indicates time dependant covariates. Models were also adjusted for the total number of cognitive assessments to correct for increased dropout of participants who started with lower cognitive scores, and include random effects for participant and a random slope with age, presuming that participants start at different baselines and vary in rate of cognitive decline. The primary coefficients of interest are those representing the number of surgery, medical, and stroke admissions before a given cognitive assessment. Those coefficients represent a cognitive step change occurring at the time of admission and persisting.

Overall, our approach presumes that participants differ in both their baseline cognitive abilities and comorbidities and their rate of decline with age. In the analysis we attempt to identify any additional cumulative change after surgery and hospital admission. We compared this approach to an alternative that treats hospital admissions as overall markers of ill health, resulting in differences in the overall cognitive trajectory without any particular impact at the time of the admission. In this model, the only variable that changes with time for a given subject is age:

Cognition_ij_=β×[1+Age_ij_+Age_ij_^2^+Age_ij_×(TotalSurgery_i_+TotalMedical_i_+TotalStroke_i_)^2^+CognitiveAssessments_i_+Covariates_i_+Covariates_i_×Age_ij_+CovariatesTD_ij_] +γ_i_×[1+Age_ij_]+ε_ij_

Because this model was inferior based on the deviance information criterion, our analyses focused on the step change model.

All models were fit using the R package MCMCglmm^30^ and custom code written in R. For final model fits we used 3000 burn-in trials followed by 100 000 iterations thinned to every 10 trials. Autocorrelation plots and Gelman-Rubin diagnostic^31^ ^32^ were used to confirm model convergence with the help of the R package coda.^33^

### Sensitivity analyses

In parallel to the models fit to the entire study sample, we analyzed a subset of 4916 participants with at least four cognitive assessments. In this population, we can better follow the cognitive trajectory in individual participants and our estimates could be less susceptible to confounding factors that cause participants to drop out of the study. In further models, we tested the impact of surgery not requiring a hospital stay of two nights, focusing on only BUPA major operations, excluding participants in high risk surgical categories (cardiac, thoracic, vascular, and intracranial neurosurgery), emergency surgery, participants with surgery before age 65, and those who had surgery after the beginning of the study period but before they completed their first cognitive assessment, and incorporating covariates.

### Interpretation of model parameter estimates

We report posterior means and bayesian 95% credible intervals. Credible intervals indicate the range of parameter estimates that are likely given the data. We chose a bayesian approach because we believe that an effect or no effect judgment based on a null hypothesis test is not the most useful or informative statistic in the context we study, and related P values are often misinterpreted.^34^ Rather, the statistic that is most relevant and most easily interpreted by clinicians and patients is the range of expected average outcomes.^35^ For example, a small but statistically significant clinical risk could be irrelevant to patients’ decisions, whereas a large but statistically inconclusive clinical risk is important. Interpreting model coefficients in terms of a range of plausible outcomes, especially estimates of the upper bound to risk, is most important to patients and clinicians.

### Missing data

To account for occasional missing demographic data, we generated 100 imputed datasets using the R package MICE,^36^ fit Markov chain Monte Carlo models to each, and computed credible intervals for each fixed effect across the imputed models.^37^

### Identifying substantial decline

As an alternative approach, we tested for the most severe (rather than average) cognitive decline outcomes. We predicted the composite cognitive scores for the final cognitive assessment (either the fourth or the fifth study wave) based on extrapolation from the first three assessments in each participant. Those with medical or surgical admissions before the third cognitive assessment, as well as those with stroke at any point during the study, were excluded, leaving 3633 participants for this approach. We fit a linear mixed effects model with age and age squared as fixed effects, and participant as a random effect with random slopes for age. Using this model, we predicted the cognitive score for each participant at their final cognitive assessment for the study, including their random intercept and slope. We subtracted these predicted cognitive scores from the actual cognitive scores at that final assessment, and then z scored these residuals across participants. In accordance with prior studies, we defined participants with z scored residuals of less than −1.96 as those experiencing “substantial decline” relative to prediction.^10^ ^38^ ^39^ We then fit a logistic regression for substantial decline as a function of having at least one surgery, at least one medical admission, or both before the final cognitive assessment, and adjusted for age at the final cognitive assessment.

### Patient and public involvement

At its initiation, participants recruited to the Whitehall II study were not involved in setting the research agenda, recruitment strategies, or study design. We did not invite participants to advise on interpretation or dissemination of our results. Nonetheless, participants receive study results through the Whitehall II website and newsletters. In 2015 the Whitehall II study randomly selected some participants to take part in a consultation exercise to guide the research agenda.

## Results

**Fig 1:**
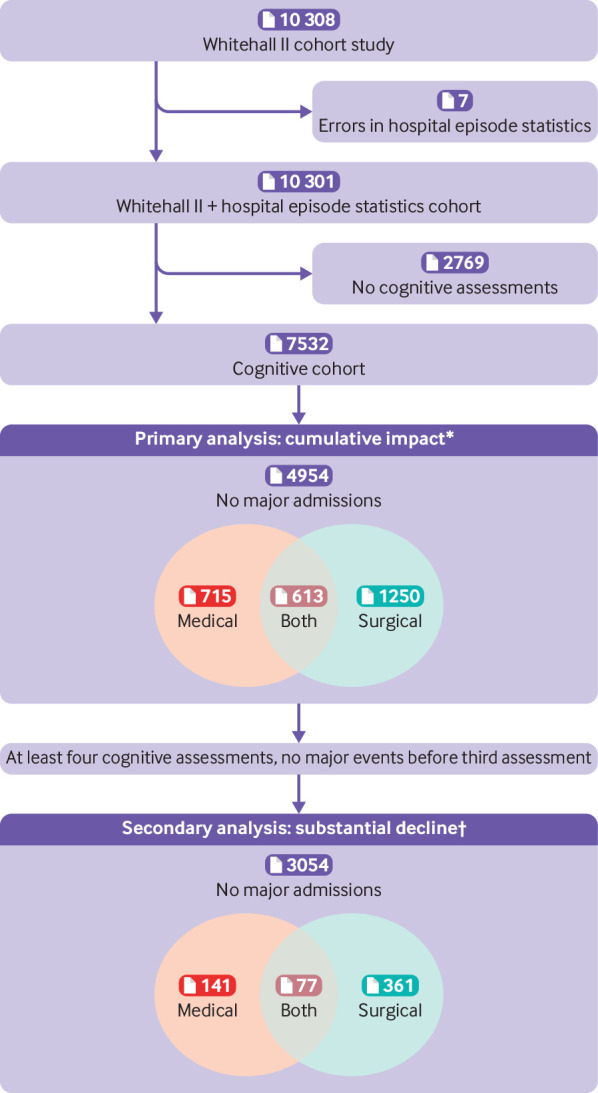
Inclusion of participants from Whitehall II cohort study and hospital episode statistics. Numbers in parentheses refer to participants, not events. *Medical admissions include stroke. Admissions after the final cognitive assessment are not included as cognitive impact cannot be assessed. †Participants with stroke excluded from this analysis

**Fig 2:**
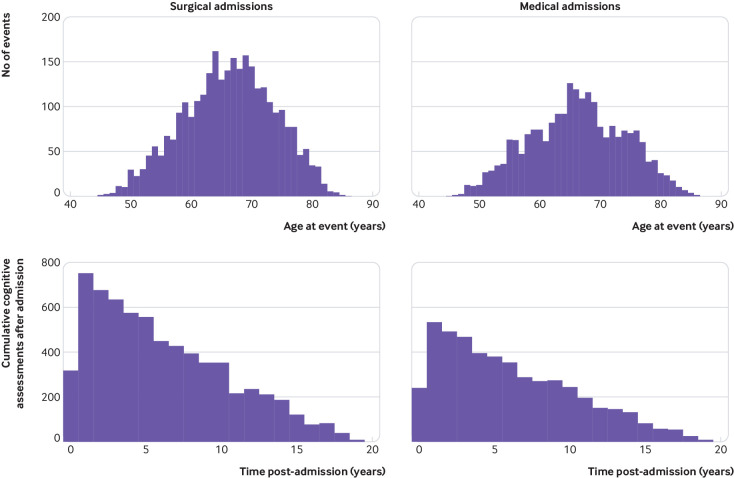
Age and duration of follow-up for surgical operations and medical admissions. (Top panel) Participant age at time of event. (Bottom panel) Cumulative follow-up duration of cognitive assessments after major events. Counts reflect both number of admissions and number of cognitive assessments and therefore each assessment is counted once for each preceding event and there are multiple assessments per participant

Figure 1 shows sample selection. Seven participants were excluded owing to errors in the hospital episode statistics database, leaving 7532 people with at least one cognitive assessment. Overall, 8982 entries were deemed “major” events and comprised 4525 operations (table 1), 4306 medical admissions, and 151 strokes. A total of 5110 entries in hospital episode statistics were made after the first cognitive assessment and before the last cognitive assessment in the study (number of admissions: 2932 operations, 2114 medical, and 64 strokes). Table 2 summarizes demographic and admission data for participants. Figure 2 summarizes the age at event and extent of follow-up after surgical and medical admissions.

**Table 1:** Classification of major surgical operations in study and those occurring before the last cognitive assessment in each participant. Values are numbers (percentages)

Major surgery	Whole study* (n=4525 admissions)	Cognitive follow-up (n=2932 admissions)
Cardiac:		
Overall	219 (4.8)	153 (5.2)
Valve	61 (1.3)	37 (1.3)
Other	158 (3.5)	116 (4.0)
Thoracic: overall	182 (4.0)	85 (2.9)
Vascular:		
Overall	139 (3.1)	84 (2.9)
Intervention	48 (1.1)	28 (1.0)
Other	91 (2.0)	56 (1.9)
Intracranial neurosurgery: overall	89 (2.0)	38 (1.3)
Major non-intracranial/non-cardiothoracic/non-vascular:		
Overall	3896 (86.1)	2572 (87.7)
General	1267 (28.0)	766 (26.1)
Orthopedic	1325 (29.3)	971 (33.1)
Other	1304 (28.8)	835 (28.5)

* Includes all surgical operations that occurred between baseline (1997-99) and March 2017, including those after the latest available cognitive assessment.

**Table 2:** Participant characteristics and events by participant category*

Baseline characteristics	Overall (n=7532)	Hospital admissions			P value†
		None (n=4954)	Any surgery (n=1863)	Medical only (n=715)	
Mean (SD) age (years)	55.5 (5.99)	55.0 (5.95)	56.6 (5.88)	56.4 (6.1)	<0.001
Women	2235 (29.7)	1465 (29.6)	599 (32.2)	171 (23.9)	<0.001
Race:					
White	6874 (91.5)	4553 (92.0)	1694 (91.1)	627 (87.9)	0.001
Non-white	645 (8.6)	394 (8.0)	165 (8.9)	86 (12.0)	
Missing	13	7	4	2	
Education level (years):					
0-12	1958 (29.1)	1297 (29.2)	501 (30.1)	160 (25.6)	0.09
13-17	3000 (44.6)	2000 (45.0)	704 (42.3)	296 (47.4)	
≥18	1771 (26.3)	1145 (25.8)	458 (27.5)	168 (26.9)	
Missing	803	512	200	91	
Maximum employment position:					
High	3413 (45.3)	2305 (46.5)	784 (42.1)	324 (45.3)	0.02
Intermediate	3219 (42.7)	2077 (41.9)	835 (44.8)	307 (42.9)	
Low	900 (12.0)	572 (11.6)	244 (13.1)	84 (11.8)	
Mean (SD) Framingham CVD risk score‡	0.20 (0.12) (n=6792)	0.19 (0.11) (n=4578)	0.21 (0.12) (n=1600)	0.22 (0.12) (n=614)	<0.001
Diabetes	889 (11.8)	495 (10.0)	277 (14.9)	117 (16.3)	<0.001
Smoking:					
Current	684 (10.0)	438 (9.7)	180 (10.7)	66 (10.4)	0.21
Former	2774 (40.7)	1803 (40.1)	716 (42.5)	255 (40.3)	
Never	3353 (49.2)	2255 (50.1)	787 (46.8)	311 (49.1)	
Missing	721	459	180	82	
Medical and surgical events:					
Mean (SD) surgical admissions	0.39 (0.85)		1.6 (1.0)		
Stroke	63 (0.8)		28 (1.5)	35 (4.9)	
Any medical admission	1328 (18)		613 (33)	715 (100)	
Mean (SD) medical admissions	0.29 (0.84)		0.60 (1.2)	1.5 (1.2)	
Mean (SD) follow-up:					
No of assessments	3.8 (1.4)	3.6 (1.5)	4.1 (1.1)	3.9 (1.2)	<0.001
Duration (years)	14.6 (5.56)	13.8 (6.25)	16.3 (3.25)	15.7 (3.85)	<0.001

* Categories are based on events occurring before each participant’s last cognitive assessment. Missing values are excluded from percentages.

† P values are from I^2^ tests (categorical) or one way analysis of variance F tests (continuous) comparing across never surgery or never medical, any surgery, and medical only categories.

‡ Averages and standard deviations are based on subset of participants owing to missing values.

We checked our a priori expectation that acute events incur a contemporaneous cognitive impact (step change model) by comparing with an alternative model in which admissions were treated as markers of chronic ill health that impact cognition continuously (rather than at the time of admission). This alternative model was inferior (change in deviance information criteria=113), so we considered only the step change model.

In the model of cumulative burden of surgical, medical, and stroke admissions (excluding covariates except the number of cognitive assessments), we found that surgical admissions were associated with only a small long term change in cognition (−0.014 units (95% credible interval −0.029 to 0.000), equivalent to 0.01 to 0.73 years of cognitive aging; table 3). Major medical admissions were associated with a significantly larger decrease in cognitive function than surgical admissions (−0.056 units (−0.073 to −0.040), equivalent to 1.0 to 1.8 years of cognitive aging, P<0.001 for contrast between surgery and medical admissions). Stroke was associated with a severe cognitive decrease (−0.52 units (−0.65 to −0.38), equivalent to 9.6 to 16 years of cognitive aging, table 3). Results with a smaller subset of participants who completed at least four cognitive assessments (n=4916) were similar (table 3).

**Table 3:** Cognitive impact of surgical admissions, medical admissions, and stroke

Admission category	Composite score	Memory	Executive function	Verbal fluency
**≥1 cognitive assessment (n=7532 participants)**				
Cognitive impact (95% CI)*				
Admissions:				
Surgical	−0.014 (−0.029 to −0.000)	−0.017 (−0.071 to 0.035)	−0.15 (−0.30 to −0.007)	−0.15 (−0.29 to −0.025)
Medical	−0.056 (−0.073 to −0.040)	−0.066 (−0.13 to −0.007)	−0.57 (−0.74 to −0.41)	−0.42 (−0.57 to −0.27)
Stroke	−0.52 (−0.65 to −0.38)	−1.0 (−1.5 to −0.52)	−5.2 (−6.5 to −3.8)	−3.5 (−4.7 to −2.3)
Years equivalent cognitive impact (95% CI)†				
Admissions:				
Surgical	0.35 (0.01 to 0.73)	0.18 (−0.35 to 0.71)	0.55 (0.03 to 1.1)	0.63 (0.10 to 1.2)
Medical	1.4 (1.0 to 1.8)	0.67 (0.07 to 1.3)	2.1 (1.5 to 2.7)	1.7 (1.1 to 2.3)
Stroke	13 (9.6 to 16)	10 (5.2 to 16)	19 (14 to 24)	14 (9.3 to 19)
**≥4 cognitive assessments (n=4916 participants)**				
Cognitive impact (95% CI)*				
Admissions:				
Surgical	−0.017 (−0.034 to −0.000)	−0.020 (−0.077 to 0.037)	−0.15 (−0.30 to 0.003)	−0.17 (−0.31 to −0.028)
Medical	−0.073 (−0.096 to −0.051)	−0.11 (−0.19 to −0.035)	−0.57 (−0.78 to −0.36)	−0.53 (−0.72 to −0.33)
Stroke	−0.65 (−0.80 to −0.49)	−1.3 (−1.9 to −0.69)	−5.2 (−6.7 to −3.8)	−3.9 (−5.3 to −2.5)
Years equivalent cognitive impact (95% CI)†				
Admissions:				
Surgical	0.41 (0.01 to 0.81)	0.20 (−0.38 to 0.78)	0.59 (−0.01 to 1.2)	0.71 (0.12 to 1.3)
Medical	1.7 (1.2 to 2.3)	1.1 (0.35 to 1.9)	2.3 (1.4 to 3.1)	2.3 (1.4 to 3.1)
Stroke	15 (12 to 19)	13 (7.0 to 19)	21 (15 to 26)	17 (11 to 22)

* Cognitive impact for composite scores are in z scored composite units per event, estimated from models adjusted for baseline effects of admissions and number of cognitive assessments, as well as random intercepts for participant and slope with age. Negative numbers indicate decline. For individual tests, units are in the scoring units on that test: questions correct (memory and executive function) or total number of words (verbal fluency).

† Years equivalent is calculated by dividing by the linear term for age in each model. Because changes with time are negative, negative numbers indicate improvement. Because tests in each domain are not on the same numeric scale, comparisons between cognitive domains should utilize the normalized “years equivalent” measure.

To contextualize the magnitude of the changes described, we also computed the change in individual cognitive tests. The post-surgical effect averaged a decrease of 0.02 recalled memory items, 0.15 fewer points on the executive function test, and 0.17 fewer words in the combined verbal and semantic fluency test (table 3). The magnitude of these average score changes are small: less than 1 point on any of the tests.

These results so far indicate the effect of surgery alone or medical admission alone. Interactions between types of admissions (surgical, medical, and stroke) were in the direction of cognitive improvement rather than a steeper decline (indicating that cognition for someone having both a surgery and a separate medical admission declined less than the summed expected decline from each separate event). Consistent with this assumption, if these interactions were not included, the estimates of cognitive decline associated with surgical (−0.010 units (−0.023 to 0.003)) or medical events (−0.048 units (−0.063 to −0.035)) would be revised downwards.

**Fig 3:**
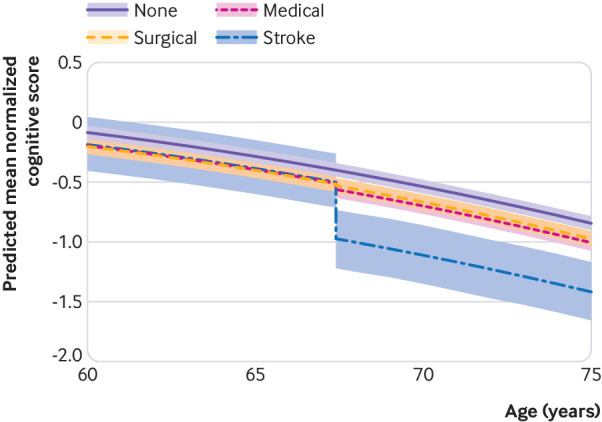
Predicted cognitive trajectory before and after admission events. Trajectories represent means and 95% credible intervals for a hypothetical average patient with a surgical or medical admission or stroke occurring at the median age (67.4 years) for first admission (or no admission). Shaded region reflects credible intervals for the impact of surgical admissions, medical admissions, and stroke rather than population variability in baseline and decline, because random effects coefficients and residuals are ignored for these prediction intervals (ie, they reflect the trajectory of a hypothetical average participant rather than the family of trajectories for a hypothetical population)

To summarize our model results, we plotted predicted cognitive trajectories for hypothetical participants (fig 3). In these predicted trajectories the overall trajectory with age was the dominant feature (change in cognitive score between ages 60 and 75 years=0.83 units, where 1 unit represents the standard deviation of the population scores at the first assessment), and a major surgical hospital admission had little impact relative to the age related trajectory. In contrast, stroke was associated with a clear effect on cognition. Our model allowed a lower baseline cognitive score in participants who would go on to have surgery, medical admissions, or stroke compared with those who did not (fig 3). These participants had a lower cognitive score at entry to the study, different from those who did not have surgery (−0.13 units (−0.18 to −0.078), equivalent to being 2.0 to 4.5 years cognitively older). Participants who had medical admissions (−0.12 units (−0.18 to −0.060), equivalent to 1.5 to 4.5 years cognitively older) also had lower baseline cognitive score, and stroke trended in that direction (−0.11 units (−0.36 to 0.12), equivalent to −3.1 to 9.0 years cognitively older), though the credible interval was wide because strokes were rare.

### Alternate modeling approach

We confirmed that similar results would be obtained with an alternate approach to model estimation using the same model structure and data, such as penalized least squares through the R package lme4.^40^ This approach gave nearly identical estimates of cognitive decline as that obtained from the MCMC approach (surgery −0.014 (95% credible interval −0.028 to 0.001); medical −0.055 (−0.073 to −0.039); stroke −0.52 (−0.65 to −0.38)).

### Sensitivity analyses

Because surgical admissions included some categories that have been specficially associated with cognitive decline,^11^ we performed sensitivity analyses excluding participants from categories of high risk surgical admissions and participants undergoing emergency surgery (table 4). Excluding participants in any one of these categories did not substantially affect the relation between surgery and cognitive scores, although excluding emergency surgery had the largest impact. Some surgical admissions that we counted as “major” according to duration of hospital stay were nonetheless associated with procedure codes that were not classified as major surgery according to BUPA scoring. However, focusing only on major operations according to BUPA (and requiring at least a two night stay), did not alter the estimate of the surgical impact (table 4).

**Table 4:** Sensitivity of cognitive impact (composite score) to surgical classes and covariates

Admission category	Years equivalent cognitive impact (95% CI)*									
	Exclude cardiac	Exclude thoracic	Exclude vascular	Exclude ICN	Exclude emergency	BUPA major†	Adjusted for LOS <2 days	Exclude surgery in under 65s	Exclude surgery pre-assessment	Adjusted for covariates‡
**≥1 cognitive assessment (n=7532 participants)**										
Admissions:										
Surgical	0.39 (0.02 to 0.77)	0.38 (−0.01 to 0.76)	0.40 (0.02 to 0.77)	0.34 (−0.02 to 0.71)	0.28 (−0.10 to 0.66)	0.32 (−0.10 to 0.72)	0.31 (−0.05 to 0.72)	0.083 (−0.43 to 0.59)	0.34 (−0.03 to 0.70)	0.34 (0.00 to 0.68)
Medical	1.3 (0.90 to 1.8)	1.4 (1.0 to 1.9)	1.4 (1.0 to 1.9)	1.4 (0.96 to 1.8)	1.4 (0.98 to 1.8)	1.4 (0.97 to 1.8)	1.4 (0.95 to 1.8)	1.3 (0.78 to 1.7)	1.4 (1.0 to 1.9)	1.2 (0.83 to 1.6)
Stroke	13 (10 to 17)	12 (8.9 to 16)	14 (10 to 17)	13 (9.5 to 16)	13 (9.6 to 16)	13 (9.4 to 16)	13 (10 to 16)	14 (10 to 17)	13 (9.5 to 16)	13 (9.5 to 16)
**≥4 cognitive assessments (n=4916 participants)**										
Admissions:										
Surgical	0.43 (0.02 to 0.84)	0.45 (0.04 to 0.87)	0.44 (0.03 to 0.85)	0.38 (−0.02 to 0.78)	0.37 (−0.05 to 0.79)	0.42 (−0.04 to 0.86)	0.32 (−0.09 to 0.74)	0.21 (−0.38 to 0.71)	0.43 (0.04 to 0.83)	0.36 (0.00 to 0.72)
Medical	1.7 (1.1 to 2.2)	1.8 (1.3 to 2.4)	1.8 (1.2 to 2.4)	1.7 (1.2 to 2.3)	1.8 (1.2 to 2.3)	1.7 (1.2 to 2.3)	1.7 (1.1 to 2.3)	1.7 (1.1 to 2.3)	1.7 (1.2 to 2.3)	1.6 (1.1 to 2.1)
Stroke	16 (12 to 19)	15 (11 to 19)	16 (12 to 20)	15 (12 to 19)	16 (12 to 19)	15 (11 to 19)	16 (12 to 19)	17 (13 to 21)	15 (12 to 19)	15 (11 to 18)

ICN=intracranial neurosurgery; LOS=length of stay.

* Years equivalent is calculated by dividing by the linear term for Age in each model. Because the changes with time are negative, negative numbers indicate improvement.

† A BUPA (private health insurance scheme) definition of a major procedure and a hospital admission of at least two nights.

‡ Models were adjusted for sex, race, education, employment level, Framingham cardiovascular disease risk, diabetes, and smoking.

We tested for a biological gradient for the surgical effect by adding cumulative minor surgical admissions as a separate factor in the model. Minor surgery (not requiring a two night stay) was not associated with change in cognition (0.13 years of cognitive aging (−0.06 to 0.32)); major surgery remained associated with a small change in cognition (0.59 years of cognitive aging (0.14 to 1.0)). Finally, we included covariates from table 2 in the analysis, assessed at baseline, and their interactions with age, using multiple imputation for missing demographic data. Results remained substantially the same (surgical event: 0.34 years of cognitive aging (0.00 to 0.68) or medical events: 1.2 years of cognitive aging (0.83 to 1.6)).

A sensitivity analysis based on the age of exposure to surgery was conducted based on the hypothesis that older participants would be more vulnerable to the cognitive effects of surgery. Next, we excluded participants with surgical admissions before age 65. Contrary to the hypothesis, these data showed a reduced impact associated with surgery at older ages (table 4).

### Incidence of substantial decline

The small average effects we report could reflect large changes in a few participants rather than small, hardly noticeable changes in each participant. Therefore, although critiqued,^15^ some prior studies of postoperative cognitive decline have focused on the number of participants with the greatest declines,^10^ ^38^ ^39^ presuming that participants who show substantial decline are more likely to experience consequences that affect quality of life. Using participants who had no medical or surgical admissions early in the study (before the third cognitive assessment) and no stroke (total n=3633 participants), we extrapolated estimated cognitive scores at the final cognitive assessment and identified participants with substantial decline relative to prediction; out of the 3633 participants,115 (3.2%) people showed substantial decline (20/361 surgery, 18/141 medical, 4/77 surgery and medical, 77/3054 neither).

Compared with participants who had no major hospital admissions, those with surgical or medical events were more likely to have substantial decline (surgical admissions odds ratio 2.3 (95% credible interval 1.4 to 3.9); medical admissions odds ratio 6.2 (3.4 to 11.0); both surgery and medical odds ratio 2.0 (0.64 to 6.0)). Participants who had only medical events were more likely to show substantial decline than participants who only had surgical events (95% credible interval for posterior density difference 1.4 to 6.1, P=0.004). Adding terms for the age at (earliest) surgery did not have a significant effect (P=0.47) if age at final assessment was included as a covariate.

## Discussion

This longitudinal study of 7532 people identified a small decrease in cognitive performance associated with surgery, equivalent to less than five months of mean cognitive decline. Hence we estimate a cognitive age of 67 years and 10 months for a participant who incurred surgery at the median age of 67 years and 5 months. To further contextualise this finding, the mean effect of a surgery is less than one standard deviation of the normal annualized decline in the population. Declines of less than one standard deviation of the age related cognitive trajectory have been suggested to lack clinical significance.^20^ We also found no evidence from the different sets of analyses, including variations in the participant population and adjustment for confounding, that on average this decrease could be greater than 12 months equivalent of cognitive aging. Major surgery was associated with less cognitive impact than major medical events or stroke although the effect was greater than for minor surgery. Indeed on average stroke was associated with a 37-fold larger and medical admissions four-fold larger cognitive effect than major surgery.

Systematic reviews of studies that largely did not consider the cognitive trajectory have suggested that any cognitive effect of surgery is detectable for six months at maximum.^9^ ^41^ Our results, however, suggest that on average major surgery might be associated with a long term change in cognition, but the mean effect is small—equivalent to less than a year of aging. Our data do suggest an increase in the odds of substantial cognitive decline after surgery, affecting 5.5% of people compared with 2.5% without major admissions. Hence while at the population level the mean effect is small, the risk of a large cognitive “hit” is about doubled, though this affects only a small percentage of the population. The threshold for our definition of substantial cognitive decline—a deviation of more than 1.96 standard deviations—has been proposed to be clinically important.^10^ ^20^

### Comparison with previous studies

A previous study did not find an effect of surgery (n=180) or major medical illness (n=119) on the cognitive trajectory when data were analyzed from the Alzheimer’s Disease Research Center (n=575).^12^ More recently, a study using four waves of data (n=431) found that incident surgery during eight years of cognitive follow-up was not associated with decline.^42^ In a larger cohort, the same authors suggested that surgery in the prior 20 years was associated with cognitive decline over the subsequent eight years^42^; however, this design cannot exclude that this effect could be attributable to lower cognitive performance before surgery contrary to the present analyses that account for these differences. Another study, of 130 participants who had surgery between cognitive assesssments (similar to our methods) in the Wisconsin Registry for Alzheimer’s Protection study (completed over nine years), found that a cumulative number of operations was associated with a decline in short term memory but not in executive function.^43^ This study did not, however, analyse other types of admissions, such as medical. A similar problem affected another study,^44^ which showed no difference in cognitive decline between patients undergoing self reported cardiac surgery or cardiac catheterization. Nonetheless, it is interesting that their estimate of 4.6 months of cognitive aging associated with cardiac surgery is closely aligned to our estimate. Multiple studies have provided estimates of a binary incidence of cognitive decline, though these have been hard to synthesize owing to varying definitions of decline,^8^ and largely these studies were unable to account for the preoperative trajectory. Systematic reviews have failed to find convincing evidence for long term effects on cognition using these binary endpoints.^8^ ^9^ ^41^ Our data are consistent with a low incidence of long term substantial cognitive decline. We were likely able to detect this low incidence based on both the large sample size and the design utilizing a predicted preoperative cognitive trajectory.

### Strengths and limitations of this study

The results from our study are robust for several reasons. First, our sample size is larger and we had longer follow-up than in previous studies, providing greater power to estimate the association between surgery and the cognitive trajectory. Second, we examined admissions for medical conditions separately from surgical admissions as considering them together could exaggerate any association with surgery. This is important given that our results show medical events to have a more profound association with the cognitive trajectory. Third, our modeling approaches allow for a better assessment of the age related cognitive trajectory than previous approaches^12^ ^42^ ^43^—for example, fitting an age^2^ term to allow for accelerated cognitive decline with aging.^28^ Our analyses also suggest that modeling a step change around the time of the event better fits the data than the longitudinal changes in the trajectory used in previous studies.^12^ ^34^ ^35^ Fourth, all events occurred over the same period as the cognitive assessments, therefore we were able to estimate cognition both before and after surgery. Some studies relied on operations occurring before the period of cognitive assessments, potentially over-estimating the association between surgery and the trajectory. Our statistical models allowed variations in individual trajectories and differences in cognitive performance before admissions. Fifth, we also examined cognitive decline in medical admissions and in participants after stroke, as well as in minor surgery, to allow inferences about the effect size of surgery and testing for a biological gradient of the effect of surgery. Sixth, we also investigated the association between major surgical and medical admissions and the incidence of substantial cognitive decline, as defined by more than 1.96 standard deviations from predicted. This result showed that surgery was associated with a doubling in the odds of substantial decline, which was smaller than for major medical admission. Seventh, postoperative cognitive improvement has been noted after surgery,^15^ and our analytic approach allowed for detection of both improvement or decline. However we found little evidence to support postoperative cognitive improvement in our results.

One surprising result from the sensitivity analyses was that in people who did not have surgery before age 65, subsequent surgical admissions were not associated with any cognitive decline. This result might reflect a selection bias in type of surgery conducted at different ages, or that longer hospital stays in younger patients are associated with surgical complications, or other factors contribute to a measurable impact. Alternatively, it could support the hypothesis that surgery at younger ages is a marker of poorer health or more aggressive disease that are associated with subsequent cognitive decline. Our study lacks data to resolve these alternatives—however, we emphasize that the mean estimates in both age groups were smaller than those seen to be clinically significant in prior studies.^10^ ^20^

Our study has several limitations. As with all observational studies we cannot ascribe causality to our findings. Indeed hospital admissions likely act as a surrogate measure for ill health, and separating the role of surgery from the underlying health condition is not possible with these data. The hospital episode statistics database also lacks data on the type of anesthesia administered, limiting our ability to assess the role of anesthesia in any long term cognitive change. As surgical admissions could serve as a surrogate measure for health, it might be most appropriate to interpret our estimated associations as an upper bound on the cognitive impact of surgery itself, with the remainder of the association ascribed to health condition necessitating surgery. The use of administrative health data to detect admissions could be considered suboptimal; however, audits show an approximate 96% accuracy for detecting events recorded in hospital episode statistics,^21^ which is likely more reliable than self report of events.^44^ Objective classification of cognitive decline might also over-estimate subjective cognitive problems,^45^ providing another reason why our results should be considered the upper bound for the impact of surgery on cognition. Finally, with any longitudinal study, we can expect loss to follow-up and this might have affected participants with the greatest impairment. With only 63 participants with stroke, we were able to show cognitive decline despite loss to follow-up. Hence loss to follow-up likely affected stroke and medical events to a greater extent than surgical events. We acknowledge that catastrophic events, such as stroke or covert stroke, can occur in the perioperative period and have cognitive consequences. These infrequent but known risks might underlie the observed increase in substantial cognitive decline. We did not seek to underestimate the impact of these surgery related events on the cognitive trajectory; however, we have labeled a stroke occurring within two weeks of surgery in this study as a surgical event and hence this would have contributed to any decline associated with surgery. These events are already established complications of surgery and are discussed during informed consent. Finally, we emphasize that our findings might not be generalizable to other groups, including those with more ethnic diversity and those with greater numbers of participants older than 70 years old, when the incidence of clinical diagnoses of cognitive decline, such as dementia, increases.

### Conclusions

Overall our data suggest that major surgery is associated with a small long term mean change in the age related cognitive trajectory, with the odds of substantial decline doubling. Hence although the mean association is small and the absolute incidence of substantial cognitive decline is low, major surgery is associated with a long term effect on cognition. This information should be conveyed to patients and be weighed against the potential health and quality of life benefits of surgery during informed consent discussions with patients.

**What is already known on this topic**

Concerns are that surgery might be associated with long term cognitive harm
Studies investigating these associations have yielded inconsistent results, partly due to methodological issues with assessment of cognition longitudinally
A major problem is the lack of attention to the cognitive trajectory, as cognitive decline accelerates with advancing age

**What this study adds**

Our study suggests that after consideration for the age related cognitive trajectory, major surgery on average is associated with a small cognitive decline equivalent to about five months of cognitive aging
The odds of substantial cognitive decline is approximately doubled after surgery, though the odds are lower than for non-surgical admissions

## Web extra

Extra material supplied by authors

## Notes

Ethical approval: This study was approved by University College London Hospital Committee on the Ethics of Human Research (reference No 85/0938), and University of Wisconsin, Madison, USA institutional review board (IRB No 2018-0658).

Data sharing: Data are publicly available through application for access to the Whitehall II database.

Transparency: The lead authors (BMK and RDS) affirms that the manuscript is an honest, accurate, and transparent account of the study being reported; that no important aspects of the study have been omitted; and that any discrepancies from the study as planned (and, if relevant, registered) have been explained.

## References

[1] Singh-Manoux A Kivimaki M Glymour MM Timing of onset of cognitive decline: results from Whitehall II prospective cohort study. BMJ 2012;344:d7622. 10.1136/bmj.d7622 22223828 PMC3281313

[2] Sabia S Marmot M Dufouil C Singh-Manoux A Smoking history and cognitive function in middle age from the Whitehall II study. Arch Intern Med 2008;168:1165-73. 10.1001/archinte.168.11.1165 18541824 PMC2696613

[3] Kaffashian S Dugravot A Elbaz A Predicting cognitive decline: a dementia risk score vs. the Framingham vascular risk scores. Neurology 2013;80:1300-6. 10.1212/WNL.0b013e31828ab370 23547265 PMC3656460

[4] Samieri C Perier MC Gaye B Association of Cardiovascular Health Level in Older Age With Cognitive Decline and Incident Dementia. JAMA 2018;320:657-64. 10.1001/jama.2018.11499 30140876 PMC6142948

[5] Levine DA Davydow DS Hough CL Langa KM Rogers MA Iwashyna TJ Functional disability and cognitive impairment after hospitalization for myocardial infarction and stroke. Circ Cardiovasc Qual Outcomes 2014;7:863-71. 10.1161/HCQ.0000000000000008 25387772 PMC4241126

[6] Bedford PD Adverse cerebral effects of anaesthesia on old people. Lancet 1955;269:259-63. 10.1016/S0140-6736(55)92689-1 13243706

[7] Rowley P Boncyk C Gaskell A What do people expect of general anaesthesia? Br J Anaesth 2017;118:486-8. 10.1093/bja/aex040 28403409

[8] Nadelson MR Sanders RD Avidan MS Perioperative cognitive trajectory in adults. Br J Anaesth 2014;112:440-51. 10.1093/bja/aet420 24384981

[9] Avidan MS Evers AS The Fallacy of Persistent Postoperative Cognitive Decline. Anesthesiology 2016;124:255-8. 10.1097/ALN.0000000000000958 26785428 PMC5839806

[10] Moller JT Cluitmans P Rasmussen LS International Study of Post-Operative Cognitive Dysfunction Long-term postoperative cognitive dysfunction in the elderly ISPOCD1 study. ISPOCD investigators. Lancet 1998;351:857-61. 10.1016/S0140-6736(97)07382-0 9525362

[11] Newman MF Kirchner JL Phillips-Bute B Neurological Outcome Research Group and the Cardiothoracic Anesthesiology Research Endeavors Investigators Longitudinal assessment of neurocognitive function after coronary-artery bypass surgery. N Engl J Med 2001;344:395-402. 10.1056/NEJM200102083440601 11172175

[12] Avidan MS Searleman AC Storandt M Long-term cognitive decline in older subjects was not attributable to noncardiac surgery or major illness. Anesthesiology 2009;111:964-70. 10.1097/ALN.0b013e3181bc9719 19786858 PMC2783989

[13] Selnes OA Grega MA Bailey MM Cognition 6 years after surgical or medical therapy for coronary artery disease. Ann Neurol 2008;63:581-90. 10.1002/ana.21382 18481292

[14] Hlatky MA Bacon C Boothroyd D Cognitive function 5 years after randomization to coronary angioplasty or coronary artery bypass graft surgery. Circulation 1997;96(Suppl):II-11-4. 9386068

[15] Cormack F Shipolini A Awad WI A meta-analysis of cognitive outcome following coronary artery bypass graft surgery. Neurosci Biobehav Rev 2012;36:2118-29. 10.1016/j.neubiorev.2012.06.002 22732162

[16] Berger M Schenning KJ Brown CH 4th Perioperative Neurotoxicity Working Group Best Practices for Postoperative Brain Health: Recommendations From the Fifth International Perioperative Neurotoxicity Working Group. Anesth Analg 2018;127:1406-13. 10.1213/ANE.0000000000003841 30303868 PMC6309612

[17] Sanders RD Avidan MS Postoperative cognitive trajectories in adults: the role of inflammatory processes. Anesthesiology 2013;118:484-6. 10.1097/ALN.0b013e3182838b67 23314169

[18] Shah FA Pike F Alvarez K Bidirectional relationship between cognitive function and pneumonia. Am J Respir Crit Care Med 2013;188:586-92. 10.1164/rccm.201212-2154OC 23848267 PMC3827700

[19] Sauër AC Nathoe HM Hendrikse J Octopus Study Group Cognitive outcomes 7.5 years after angioplasty compared with off-pump coronary bypass surgery. Ann Thorac Surg 2013;96:1294-300. 10.1016/j.athoracsur.2013.05.001 23866798

[20] Singh-Manoux A Kivimäki M The importance of cognitive aging for understanding dementia. Age (Dordr) 2010;32:509-12. 10.1007/s11357-010-9147-7 20454932 PMC2980594

[21] Burns EM Rigby E Mamidanna R Systematic review of discharge coding accuracy. J Public Health (Oxf) 2012;34:138-48. 10.1093/pubmed/fdr054 21795302 PMC3285117

[22] Devereaux PJ Mrkobrada M Sessler DI POISE-2 Investigators Aspirin in patients undergoing noncardiac surgery. N Engl J Med 2014;370:1494-503. 10.1056/NEJMoa1401105 24679062

[23] Devereaux PJ Sessler DI Leslie K POISE-2 Investigators Clonidine in patients undergoing noncardiac surgery. N Engl J Med 2014;370:1504-13. 10.1056/NEJMoa1401106 24679061

[24] Devereaux PJ Yang H Yusuf S POISE Study Group Effects of extended-release metoprolol succinate in patients undergoing non-cardiac surgery (POISE trial): a randomised controlled trial. Lancet 2008;371:1839-47. 10.1016/S0140-6736(08)60601-7 18479744

[25] Sutton R Bann S Brooks M Sarin S The Surgical Risk Scale as an improved tool for risk-adjusted analysis in comparative surgical audit. Br J Surg 2002;89:763-8. 10.1046/j.1365-2168.2002.02080.x 12027988

[26] Venkatesan S Myles PR Manning HJ Cohort study of preoperative blood pressure and risk of 30-day mortality after elective non-cardiac surgery. Br J Anaesth 2017;119:65-77. 10.1093/bja/aex056 28633374

[27] Singh-Manoux A Dugravot A Brunner E Interleukin-6 and C-reactive protein as predictors of cognitive decline in late midlife. Neurology 2014;83:486-93. 10.1212/WNL.0000000000000665 24991031 PMC4141998

[28] Rusmaully J Dugravot A Moatti JP Contribution of cognitive performance and cognitive decline to associations between socioeconomic factors and dementia: A cohort study. PLoS Med 2017;14:e1002334. 10.1371/journal.pmed.1002334 28650972 PMC5484463

[29] D’Agostino RB Sr Vasan RS Pencina MJ General cardiovascular risk profile for use in primary care: the Framingham Heart Study. Circulation 2008;117:743-53. 10.1161/CIRCULATIONAHA.107.699579 18212285

[30] Hadfield JD MCMC methods for multi-response generalized linear mixed models: the MCMCglmm R package. J Stat Softw 2010;33:1-22 10.18637/jss.v033.i02 .20808728

[31] Gelman A Rubin DB Inference from iterative simulation using multiple sequences. Stat Sci 1992;7:457-72 10.1214/ss/1177011136 .

[32] Brooks SP Gelman A General methods for monitoring convergence of iterative simulations. J Comput Graph Stat 1998;7:434-55.

[33] Plummer M Best N Cowles K Vines K CODA: convergence diagnosis and output analysis for MCMC. R News 2006;6:7-11.

[34] Ioannidis JPA The proposal to lower p value thresholds to. 005. JAMA 2018;319:1429-30. 10.1001/jama.2018.1536 29566133

[35] Lewis RJ Angus DC Time for Clinicians to Embrace Their Inner Bayesian?: Reanalysis of Results of a Clinical Trial of Extracorporeal Membrane Oxygenation. JAMA 2018;320:2208-10. 10.1001/jama.2018.16916 30347047

[36] van Buuren S Groothuis-Oudshoorn K mice: Multivariate imputation by chained equations in R. J Stat Softw 2011;45:1-68 10.18637/jss.v045.i03 .

[37] Zhou X Reiter JP A note on Bayesian inference after multiple imputation. Am Stat 2010;64:159-63 10.1198/tast.2010.09109 .

[38] Abildstrom H Rasmussen LS Rentowl P International Study of Post-Operative Cognitive Dysfunction Cognitive dysfunction 1-2 years after non-cardiac surgery in the elderly. ISPOCD group. Acta Anaesthesiol Scand 2000;44:1246-51. 10.1034/j.1399-6576.2000.441010.x 11065205

[39] Rasmussen LS Siersma VD ISPOCD GROUP Postoperative cognitive dysfunction: true deterioration versus random variation. Acta Anaesthesiol Scand 2004;48:1137-43. 10.1111/j.1399-6576.2004.00502.x 15352960

[40] Bates D Mächler M Bolker B Walker S Fitting linear mixed-effects models using lme4. J Stat Softw 2015;67:1-48. 10.18637/jss.v067.i01 .

[41] Avidan MS Evers AS Review of clinical evidence for persistent cognitive decline or incident dementia attributable to surgery or general anesthesia. J Alzheimers Dis 2011;24:201-16. 10.3233/JAD-2011-101680 21447878

[42] Schulte PJ Roberts RO Knopman DS Association between exposure to anaesthesia and surgery and long-term cognitive trajectories in older adults: report from the Mayo Clinic Study of Aging. Br J Anaesth 2018;121:398-405. 10.1016/j.bja.2018.05.060 30032878 PMC6200109

[43] Bratzke LC Koscik RL Schenning KJ Cognitive decline in the middle-aged after surgery and anaesthesia: results from the Wisconsin Registry for Alzheimer’s Prevention cohort. Anaesthesia 2018;73:549-55. 10.1111/anae.14216 29468634 PMC5978420

[44] Whitlock EL Diaz-Ramirez LG Smith AK Boscardin WJ Avidan MS Glymour MM Cognitive change after cardiac surgery versus cardiac catheterization: a population-based study. Ann Thorac Surg 2019;107:1119-25. 10.1016/j.athoracsur.2018.10.021 30578068 PMC6707506

[45] Deiner S Liu X Lin HM Subjective cognitive complaints in patients undergoing major non-cardiac surgery: a prospective single centre cohort trial. Br J Anaesth 2019;122:742-50. 10.1016/j.bja.2019.02.027 31003631 PMC6676774

