# Supplementary material for: Association between major surgical admissions and the cognitive trajectory: 19 year follow-up of Whitehall II cohort study
Source: BMJ. 2019 Aug 7;366:l4466. doi: 10.1136/bmj.l4466 (PMC6683971; doi:10.1136/bmj.l4466)
Supplement: Supplementary file 1 — Supplementary information: appendix 1 [file krab049203.ww1.pdf]

OPCS Code	Surgical_Type	BUPA surgical severity rating score (NB. BUPA major are scores greater than or equal to 3)	Emergency Surgery
K104	cardiac_surgery	5	
K221	cardiac_surgery	5	
K234	cardiac_surgery	5	
K334	cardiac_surgery	5	
K401	cardiac_surgery	5	
K402	cardiac_surgery	5	
K403	cardiac_surgery	5	
K404	cardiac_surgery	5	
K411	cardiac_surgery	5	
K412	cardiac_surgery	5	
K413	cardiac_surgery	5	
K442	cardiac_surgery	5	
K451	cardiac_surgery	5	
K452	cardiac_surgery	5	
K453	cardiac_surgery	5	
K454	cardiac_surgery	5	
K455	cardiac_surgery	5	
K668	cardiac_surgery	5	
K679	cardiac_surgery	5	
K681	cardiac_surgery	5	
K682	cardiac_surgery	5	
K688	cardiac_surgery	5	
K689	cardiac_surgery	5	
K698	cardiac_surgery	5	
L031	cardiac_surgery	5	
L191	cardiac_surgery	5	
L202	cardiac_surgery	5	
K252	cardiac_surgery_valve	5	
K253	cardiac_surgery_valve	5	
K254	cardiac_surgery_valve	5	
K255	cardiac_surgery_valve	5	
K259	cardiac_surgery_valve	5	
K262	cardiac_surgery_valve	5	
K263	cardiac_surgery_valve	5	
K264	cardiac_surgery_valve	5	
K268	cardiac_surgery_valve	5	
K302	cardiac_surgery_valve	5	
D011	ENT_surgery	2	
D012	ENT_surgery	2	

D021	ENT_surgery	2
D034	ENT_surgery	2
D041	ENT_surgery	2
D063	ENT_surgery	4
D078	ENT_surgery	2
D102	ENT_surgery	4
D103	ENT_surgery	4
D104	ENT_surgery	4
D105	ENT_surgery	4
D106	ENT_surgery	4
D121	ENT_surgery	4
D122	ENT_surgery	4
D124	ENT_surgery	4
D128	ENT_surgery	4
D132	ENT_surgery	3
D141	ENT_surgery	3
D142	ENT_surgery	3
D148	ENT_surgery	3
D151	ENT_surgery	3
D152	ENT_surgery	3
D153	ENT_surgery	3
D171	ENT_surgery	3
D172	ENT_surgery	3
D241	ENT_surgery	3
D282	ENT_surgery	3
D288	ENT_surgery	2
E024	ENT_surgery	2
E031	ENT_surgery	2
E036	ENT_surgery	2
E038	ENT_surgery	2
E041	ENT_surgery	2
E042	ENT_surgery	2
E048	ENT_surgery	2
E051	ENT_surgery	2
E052	ENT_surgery	2
E058	ENT_surgery	2
E061	ENT_surgery	1
E062	ENT_surgery	1
E063	ENT_surgery	2
E064	ENT_surgery	2
E068	ENT_surgery	1
E069	ENT_surgery	1
E081	ENT_surgery	2
E085	ENT_surgery	2

E091	ENT_surgery	1
E093	ENT_surgery	1
E094	ENT_surgery	1
E098	ENT_surgery	1
E101	ENT_surgery	1
E133	ENT_surgery	2
E134	ENT_surgery	2
E135	ENT_surgery	2
E136	ENT_surgery	2
E141	ENT_surgery	4
E142	ENT_surgery	4
E144	ENT_surgery	4
E149	ENT_surgery	4
E151	ENT_surgery	4
E158	ENT_surgery	4
E173	ENT_surgery	4
E178	ENT_surgery	4
E179	ENT_surgery	4
E192	ENT_surgery	5
E201	ENT_surgery	5
E232	ENT_surgery	5
E242	ENT_surgery	5
E243	ENT_surgery	5
E248	ENT_surgery	5
E251	ENT_surgery	3
E252	ENT_surgery	3
E253	ENT_surgery	2
E259	ENT_surgery	2
E272	ENT_surgery	4
E274	ENT_surgery	3
E275	ENT_surgery	3
E276	ENT_surgery	4
E278	ENT_surgery	4
E333	ENT_surgery	4
E335	ENT_surgery	4
E338	ENT_surgery	4
E341	ENT_surgery	4
E342	ENT_surgery	4
E348	ENT_surgery	4
E352	ENT_surgery	4
E358	ENT_surgery	4
E361	ENT_surgery	4
E368	ENT_surgery	4
E369	ENT_surgery	4

E371	ENT_surgery	4
E381	ENT_surgery	4
E401	ENT_surgery	4
E423	ENT_surgery	4
E424	ENT_surgery	4
E426	ENT_surgery	4
E427	ENT_surgery	4
E438	ENT_surgery	3
E659	ENT_surgery	2
F021	ENT_surgery	1
F053	ENT_surgery	1
F062	ENT_surgery	1
F091	ENT_surgery	1
F094	ENT_surgery	1
F231	ENT_surgery	3
F241	ENT_surgery	3
F265	ENT_surgery	2
F281	ENT_surgery	4
F324	ENT_surgery	4
F328	ENT_surgery	4
F341	ENT_surgery	4
F344	ENT_surgery	4
F347	ENT_surgery	4
F348	ENT_surgery	4
F349	ENT_surgery	4
F362	ENT_surgery	4
F363	ENT_surgery	3
F381	ENT_surgery	3
F382	ENT_surgery	3
F392	ENT_surgery	4
F421	ENT_surgery	2
F428	ENT_surgery	2
F441	ENT_surgery	4
F442	ENT_surgery	4
F443	ENT_surgery	4
F444	ENT_surgery	4
F451	ENT_surgery	4
F452	ENT_surgery	4
F461	ENT_surgery	4
F468	ENT_surgery	4
F481	ENT_surgery	2
F488	ENT_surgery	2
F512	ENT_surgery	2
F633	ENT_surgery	2

B081	general_surgery	3
B082	general_surgery	3
B083	general_surgery	3
B084	general_surgery	3
B085	general_surgery	3
B086	general_surgery	3
B089	general_surgery	3
B101	general_surgery	3
B122	general_surgery	3
B128	general_surgery	3
B144	general_surgery	3
B145	general_surgery	3
B149	general_surgery	3
B163	general_surgery	3
B181	general_surgery	5
B201	general_surgery	5
B223	general_surgery	5
B252	general_surgery	4
B272	general_surgery	4
B273	general_surgery	4
B274	general_surgery	4
B275	general_surgery	4
B276	general_surgery	4
B278	general_surgery	4
B279	general_surgery	4
B281	general_surgery	4
B282	general_surgery	4
B283	general_surgery	4
B284	general_surgery	4
B285	general_surgery	4
B287	general_surgery	4
B288	general_surgery	4
B298	general_surgery	4
B301	general_surgery	4
B302	general_surgery	4
B303	general_surgery	4
B304	general_surgery	4
B311	general_surgery	4
B318	general_surgery	4
B321	general_surgery	2
B322	general_surgery	2
B328	general_surgery	2
B329	general_surgery	2
B331	general_surgery	2

B332	general_surgery	2
B342	general_surgery	2
B354	general_surgery	2
B358	general_surgery	2
B361	general_surgery	2
B374	general_surgery	3
B375	general_surgery	3
G011	general_surgery	5
G013	general_surgery	5
G029	general_surgery	5
G031	general_surgery	5
G039	general_surgery	5
G091	general_surgery	4
G144	general_surgery	2
G151	general_surgery	2
G152	general_surgery	2
G153	general_surgery	2
G154	general_surgery	2
G156	general_surgery	2
G157	general_surgery	2
G158	general_surgery	2
G169	general_surgery	2
G184	general_surgery	3
G199	general_surgery	3
G211	general_surgery	2
G214	general_surgery	2
G215	general_surgery	2
G232	general_surgery	5
G233	general_surgery	5
G241	general_surgery	5
G243	general_surgery	5
G275	general_surgery	5
G278	general_surgery	5
G279	general_surgery	5
G281	general_surgery	5
G283	general_surgery	5
G285	general_surgery	5
G292	general_surgery	5
G308	general_surgery	5
G321	general_surgery	5
G331	general_surgery	5
G334	general_surgery	4
G341	general_surgery	3
G342	general_surgery	3

G344	general_surgery	3
G345	general_surgery	2
G351	general_surgery	5
G352	general_surgery	5
G387	general_surgery	4
G433	general_surgery	3
G434	general_surgery	3
G435	general_surgery	3
G436	general_surgery	3
G437	general_surgery	3
G438	general_surgery	3
G441	general_surgery	3
G442	general_surgery	3
G443	general_surgery	3
G445	general_surgery	3
G446	general_surgery	3
G448	general_surgery	3
G449	general_surgery	3
G451	general_surgery	3
G452	general_surgery	3
G458	general_surgery	3
G459	general_surgery	3
G478	general_surgery	3
G521	general_surgery	5
G522	general_surgery	5
G523	general_surgery	5
G532	general_surgery	5
G543	general_surgery	4
G584	general_surgery	5
G601	general_surgery	3
G608	general_surgery	5
G612	general_surgery	5
G693	general_surgery	5
G694	general_surgery	5
G698	general_surgery	5
G699	general_surgery	5
G701	general_surgery	4
G702	general_surgery	5
G715	general_surgery	5
G722	general_surgery	5
G725	general_surgery	5
G734	general_surgery	5
G738	general_surgery	5
G742	general_surgery	5

G743	general_surgery	5	
G748	general_surgery	5	
G749	general_surgery	5	
G751	general_surgery	4	
G753	general_surgery	4	
G758	general_surgery	4	
G763	general_surgery	5	
G784	general_surgery	4	
G788	general_surgery	4	
G802	general_surgery	1	
G803	general_surgery	1	
H011	general_surgery	3	emergency
H012	general_surgery	3	emergency
H013	general_surgery	3	emergency
H019	general_surgery	3	emergency
H021	general_surgery	3	
H024	general_surgery	3	
H028	general_surgery	3	
H029	general_surgery	3	
H031	general_surgery	3	
H041	general_surgery	5	
H051	general_surgery	5	
H053	general_surgery	5	
H061	general_surgery	4	
H062	general_surgery	4	
H068	general_surgery	4	
H069	general_surgery	4	
H071	general_surgery	4	
H072	general_surgery	4	
H073	general_surgery	4	
H074	general_surgery	4	
H075	general_surgery	4	
H078	general_surgery	4	
H079	general_surgery	4	
H083	general_surgery	4	
H084	general_surgery	4	
H091	general_surgery	4	
H092	general_surgery	4	
H093	general_surgery	4	
H094	general_surgery	4	
H095	general_surgery	4	
H099	general_surgery	4	
H101	general_surgery	4	
H102	general_surgery	4	

H103	general_surgery	4
H104	general_surgery	4
H105	general_surgery	4
H109	general_surgery	4
H112	general_surgery	4
H114	general_surgery	4
H115	general_surgery	4
H119	general_surgery	4
H122	general_surgery	4
H151	general_surgery	4
H152	general_surgery	4
H153	general_surgery	4
H154	general_surgery	3
H157	general_surgery	4
H201	general_surgery	2
H202	general_surgery	2
H206	general_surgery	2
H208	general_surgery	2
H214	general_surgery	2
H221	general_surgery	2
H228	general_surgery	2
H229	general_surgery	2
H231	general_surgery	2
H232	general_surgery	2
H233	general_surgery	2
H238	general_surgery	2
H239	general_surgery	2
H243	general_surgery	2
H251	general_surgery	2
H259	general_surgery	2
H262	general_surgery	2
H278	general_surgery	2
H281	general_surgery	2
H289	general_surgery	2
H298	general_surgery	4
H331	general_surgery	4
H332	general_surgery	4
H333	general_surgery	4
H334	general_surgery	4
H335	general_surgery	4
H336	general_surgery	3
H338	general_surgery	3
H354	general_surgery	3
H361	general_surgery	3

H368	general_surgery	3
H402	general_surgery	2
H411	general_surgery	3
H412	general_surgery	2
H413	general_surgery	2
H414	general_surgery	3
H426	general_surgery	3
H441	general_surgery	2
H443	general_surgery	2
H444	general_surgery	1
H448	general_surgery	1
H481	general_surgery	1
H482	general_surgery	1
H498	general_surgery	1
H511	general_surgery	2
H513	general_surgery	2
H519	general_surgery	2
H523	general_surgery	2
H524	general_surgery	2
H528	general_surgery	2
H538	general_surgery	2
H541	general_surgery	1
H548	general_surgery	2
H551	general_surgery	2
H553	general_surgery	2
H554	general_surgery	2
H558	general_surgery	2
H561	general_surgery	1
H562	general_surgery	1
H564	general_surgery	1
H568	general_surgery	1
H569	general_surgery	1
H581	general_surgery	2
H582	general_surgery	3
H602	general_surgery	3
H625	general_surgery	3
H626	general_surgery	3
H662	general_surgery	3
J021	general_surgery	5
J022	general_surgery	5
J023	general_surgery	5
J024	general_surgery	5
J032	general_surgery	5
J082	general_surgery	3

J091	general_surgery	3
J101	general_surgery	3
J103	general_surgery	3
J117	general_surgery	3
J121	general_surgery	5
J123	general_surgery	4
J124	general_surgery	4
J131	general_surgery	4
J132	general_surgery	4
J141	general_surgery	4
J168	general_surgery	5
J182	general_surgery	4
J183	general_surgery	4
J185	general_surgery	4
J188	general_surgery	4
J189	general_surgery	4
J192	general_surgery	4
J193	general_surgery	4
J211	general_surgery	4
J212	general_surgery	4
J241	general_surgery	4
J292	general_surgery	4
J305	general_surgery	4
J381	general_surgery	3
J382	general_surgery	3
J388	general_surgery	3
J389	general_surgery	3
J398	general_surgery	3
J401	general_surgery	3
J402	general_surgery	3
J403	general_surgery	3
J404	general_surgery	3
J406	general_surgery	3
J408	general_surgery	3
J411	general_surgery	3
J412	general_surgery	3
J413	general_surgery	3
J421	general_surgery	3
J428	general_surgery	3
J431	general_surgery	3
J433	general_surgery	3
J438	general_surgery	3
J439	general_surgery	3
J449	general_surgery	3

J462	general_surgery	3
J471	general_surgery	3
J475	general_surgery	3
J478	general_surgery	3
J502	general_surgery	3
J562	general_surgery	5
J563	general_surgery	5
J571	general_surgery	5
J575	general_surgery	5
J578	general_surgery	5
J594	general_surgery	5
J611	general_surgery	5
J614	general_surgery	5
J619	general_surgery	5
J662	general_surgery	4
J664	general_surgery	4
J671	general_surgery	4
J673	general_surgery	4
J678	general_surgery	4
J692	general_surgery	4
J749	general_surgery	2
T202	general_surgery	2
T203	general_surgery	2
T204	general_surgery	2
T208	general_surgery	2
T209	general_surgery	2
T212	general_surgery	2
T213	general_surgery	2
T219	general_surgery	2
T223	general_surgery	2
T242	general_surgery	2
T243	general_surgery	2
T248	general_surgery	2
T249	general_surgery	2
T251	general_surgery	2
T252	general_surgery	2
T253	general_surgery	2
T258	general_surgery	2
T259	general_surgery	2
T262	general_surgery	2
T271	general_surgery	2
T272	general_surgery	2
T273	general_surgery	2
T283	general_surgery	2

T288	general_surgery	2
T301	general_surgery	5
T302	general_surgery	4
T309	general_surgery	4
T313	general_surgery	2
T315	general_surgery	2
T331	general_surgery	3
T343	general_surgery	4
T348	general_surgery	4
T361	general_surgery	5
T364	general_surgery	4
T391	general_surgery	4
T411	general_surgery	3
T412	general_surgery	3
T413	general_surgery	3
T419	general_surgery	3
T423	general_surgery	3
T431	general_surgery	3
T432	general_surgery	3
T439	general_surgery	3
T451	general_surgery	3
T452	general_surgery	3
T453	general_surgery	3
T454	general_surgery	3
T458	general_surgery	3
T463	general_surgery	3
T468	general_surgery	3
T469	general_surgery	3
T483	general_surgery	3
T851	general_surgery	2
T852	general_surgery	2
T855	general_surgery	2
T858	general_surgery	2
T861	general_surgery	2
T862	general_surgery	2
T867	general_surgery	2
T868	general_surgery	2
T872	general_surgery	2
T873	general_surgery	2
T875	general_surgery	2
T877	general_surgery	2
T878	general_surgery	2
T879	general_surgery	2
T911	general_surgery	2

T929	general_surgery	2
T973	general_surgery	2
T982	general_surgery	2
M523	Gynaecology_surgery	3
M533	Gynaecology_surgery	3
M536	Gynaecology_surgery	3
M538	Gynaecology_surgery	3
M554	Gynaecology_surgery	3
M563	Gynaecology_surgery	2
P052	Gynaecology_surgery	2
P054	Gynaecology_surgery	2
P061	Gynaecology_surgery	2
P063	Gynaecology_surgery	2
P064	Gynaecology_surgery	2
P091	Gynaecology_surgery	2
P092	Gynaecology_surgery	2
P131	Gynaecology_surgery	2
P202	Gynaecology_surgery	3
P205	Gynaecology_surgery	3
P229	Gynaecology_surgery	3
P231	Gynaecology_surgery	3
P232	Gynaecology_surgery	3
P233	Gynaecology_surgery	3
P234	Gynaecology_surgery	3
P237	Gynaecology_surgery	3
P242	Gynaecology_surgery	3
P247	Gynaecology_surgery	3
P248	Gynaecology_surgery	3
P255	Gynaecology_surgery	3
P298	Gynaecology_surgery	3
Q013	Gynaecology_surgery	1
Q023	Gynaecology_surgery	1
Q028	Gynaecology_surgery	1
Q032	Gynaecology_surgery	1
Q033	Gynaecology_surgery	1
Q035	Gynaecology_surgery	1
Q038	Gynaecology_surgery	1
Q039	Gynaecology_surgery	1
Q072	Gynaecology_surgery	3
Q074	Gynaecology_surgery	3
Q075	Gynaecology_surgery	3
Q079	Gynaecology_surgery	3
Q083	Gynaecology_surgery	3
Q088	Gynaecology_surgery	3

Q089	Gynaecology_surgery	3
Q103	Gynaecology_surgery	2
Q113	Gynaecology_surgery	2
Q121	Gynaecology_surgery	1
Q151	Gynaecology_surgery	3
Q171	Gynaecology_surgery	3
Q174	Gynaecology_surgery	3
Q178	Gynaecology_surgery	3
Q181	Gynaecology_surgery	3
Q188	Gynaecology_surgery	3
Q189	Gynaecology_surgery	3
Q202	Gynaecology_surgery	2
Q221	Gynaecology_surgery	3
Q223	Gynaecology_surgery	3
Q228	Gynaecology_surgery	3
Q231	Gynaecology_surgery	3
Q235	Gynaecology_surgery	3
Q236	Gynaecology_surgery	3
Q241	Gynaecology_surgery	3
Q243	Gynaecology_surgery	3
Q352	Gynaecology_surgery	2
Q353	Gynaecology_surgery	3
Q432	Gynaecology_surgery	3
Q438	Gynaecology_surgery	3
Q472	Gynaecology_surgery	3
Q491	Gynaecology_surgery	3
Q552	Gynaecology_surgery	1
Q558	Gynaecology_surgery	1
Q559	Gynaecology_surgery	1
A021	intracranial_neurosurgery	5
A022	intracranial_neurosurgery	5
A023	intracranial_neurosurgery	5
A024	intracranial_neurosurgery	5
A028	intracranial_neurosurgery	5
A029	intracranial_neurosurgery	5
A038	intracranial_neurosurgery	5
A039	intracranial_neurosurgery	5
A041	intracranial_neurosurgery	4
A042	intracranial_neurosurgery	5
A054	intracranial_neurosurgery	5
A081	intracranial_neurosurgery	4
A083	intracranial_neurosurgery	4
A086	intracranial_neurosurgery	4
A089	intracranial_neurosurgery	4

A104	intracranial_neurosurgery	4
A124	intracranial_neurosurgery	4
A125	intracranial_neurosurgery	4
A134	intracranial_neurosurgery	4
A161	intracranial_neurosurgery	4
A201	intracranial_neurosurgery	4
A203	intracranial_neurosurgery	4
A255	intracranial_neurosurgery	5
A295	intracranial_neurosurgery	5
A296	intracranial_neurosurgery	5
A323	intracranial_neurosurgery	4
A324	intracranial_neurosurgery	4
A326	intracranial_neurosurgery	4
A331	intracranial_neurosurgery	4
A333	intracranial_neurosurgery	4
A381	intracranial_neurosurgery	5
A383	intracranial_neurosurgery	5
A384	intracranial_neurosurgery	5
A386	intracranial_neurosurgery	5
A388	intracranial_neurosurgery	5
A389	intracranial_neurosurgery	5
A398	intracranial_neurosurgery	5
A411	intracranial_neurosurgery	5
B012	intracranial_neurosurgery	5
B041	intracranial_neurosurgery	5
L332	intracranial_neurosurgery	5
L338	intracranial_neurosurgery	5
L351	intracranial_neurosurgery	5
L352	intracranial_neurosurgery	4
O052	intracranial_neurosurgery	4
V031	intracranial_neurosurgery	5
V033	intracranial_neurosurgery	5
V054	intracranial_neurosurgery	4
V058	intracranial_neurosurgery	4
V093	intracranial_neurosurgery	5
O061	Orthopedic	4
O071	Orthopedic	4
O172	Orthopedic	4
O178	Orthopedic	4
O181	Orthopedic	4
O211	Orthopedic	4
O291	Orthopedic	4
R121	Obstetric_surgery	4
R151	Obstetric_surgery	3

R229	Obstetric_surgery	3
R323	Obstetric_surgery	4
C012	opthalmic_surgery	2
C013	opthalmic_surgery	2
C061	opthalmic_surgery	2
C063	opthalmic_surgery	3
C115	opthalmic_surgery	4
C121	opthalmic_surgery	2
C131	opthalmic_surgery	2
C132	opthalmic_surgery	2
C134	opthalmic_surgery	2
C142	opthalmic_surgery	2
C148	opthalmic_surgery	2
C152	opthalmic_surgery	2
C171	opthalmic_surgery	2
C253	opthalmic_surgery	2
C254	opthalmic_surgery	3
C321	opthalmic_surgery	3
C322	opthalmic_surgery	3
C411	opthalmic_surgery	2
C438	opthalmic_surgery	2
C441	opthalmic_surgery	2
C462	opthalmic_surgery	2
C463	opthalmic_surgery	2
C465	opthalmic_surgery	2
C543	opthalmic_surgery	2
C544	opthalmic_surgery	2
C545	opthalmic_surgery	2
C548	opthalmic_surgery	2
C549	opthalmic_surgery	2
C553	opthalmic_surgery	2
C578	opthalmic_surgery	2
C601	opthalmic_surgery	2
C605	opthalmic_surgery	2
C608	opthalmic_surgery	2
C612	opthalmic_surgery	2
C623	opthalmic_surgery	2
C678	opthalmic_surgery	2
C691	opthalmic_surgery	2
C694	opthalmic_surgery	2
C712	opthalmic_surgery	2
C719	opthalmic_surgery	2
C751	opthalmic_surgery	2
C791	opthalmic_surgery	2

C792	opthalmic_surgery	2
C793	opthalmic_surgery	2
C794	opthalmic_surgery	2
C795	opthalmic_surgery	2
C797	opthalmic_surgery	2
C798	opthalmic_surgery	2
C799	opthalmic_surgery	2
C801	opthalmic_surgery	2
C802	opthalmic_surgery	2
C803	opthalmic_surgery	2
C822	opthalmic_surgery	2
C823	opthalmic_surgery	2
C824	opthalmic_surgery	2
C828	opthalmic_surgery	2
C841	opthalmic_surgery	2
C848	opthalmic_surgery	2
T621	Orthopedic	2
T622	Orthopedic	2
T628	Orthopedic	2
T642	Orthopedic	2
T652	Orthopedic	2
T673	Orthopedic	2
T676	Orthopedic	2
T678	Orthopedic	2
T679	Orthopedic	2
T688	Orthopedic	2
T691	Orthopedic	2
T702	Orthopedic	2
T705	Orthopedic	2
T723	Orthopedic	2
T742	Orthopedic	2
T744	Orthopedic	2
T749	Orthopedic	2
T761	Orthopedic	2
T772	Orthopedic	2
T773	Orthopedic	2
T779	Orthopedic	2
T791	Orthopedic	2
T792	Orthopedic	2
T798	Orthopedic	2
T799	Orthopedic	2
T811	Orthopedic	2
T818	Orthopedic	2
W032	Orthopedic	2

W035	Orthopedic	2	
W036	Orthopedic	2	
W038	Orthopedic	2	
W042	Orthopedic	2	
W043	Orthopedic	2	
W051	Orthopedic	2	
W059	Orthopedic	2	
W063	Orthopedic	2	
W066	Orthopedic	2	
W068	Orthopedic	2	
W069	Orthopedic	2	
W072	Orthopedic	2	
W082	Orthopedic	2	
W083	Orthopedic	2	
W085	Orthopedic	2	
W089	Orthopedic	2	
W091	Orthopedic	2	
W097	Orthopedic	2	
W102	Orthopedic	2	
W132	Orthopedic	2	
W133	Orthopedic	2	
W141	Orthopedic	2	
W146	Orthopedic	2	
W151	Orthopedic	2	
W152	Orthopedic	2	
W153	Orthopedic	2	
W156	Orthopedic	2	
W157	Orthopedic	2	
W158	Orthopedic	2	
W159	Orthopedic	2	
W164	Orthopedic	2	
W168	Orthopedic	2	
W169	Orthopedic	2	
W191	Orthopedic	4	emergency
W192	Orthopedic	4	emergency
W193	Orthopedic	4	emergency
W194	Orthopedic	3	emergency
W195	Orthopedic	3	emergency
W196	Orthopedic	3	emergency
W198	Orthopedic	3	emergency
W199	Orthopedic	3	emergency
W201	Orthopedic	4	emergency
W202	Orthopedic	4	emergency
W205	Orthopedic	3	emergency

W208	Orthopedic	3	emergency
W209	Orthopedic	3	emergency
W212	Orthopedic	3	emergency
W214	Orthopedic	3	emergency
W215	Orthopedic	3	emergency
W222	Orthopedic	3	emergency
W228	Orthopedic	3	emergency
W229	Orthopedic	3	emergency
W231	Orthopedic	3	emergency
W232	Orthopedic	3	emergency
W241	Orthopedic	4	emergency
W242	Orthopedic	4	emergency
W243	Orthopedic	4	emergency
W244	Orthopedic	4	emergency
W246	Orthopedic	3	emergency
W248	Orthopedic	3	emergency
W249	Orthopedic	3	emergency
W251	Orthopedic	3	emergency
W253	Orthopedic	3	emergency
W259	Orthopedic	3	emergency
W262	Orthopedic	3	emergency
W264	Orthopedic	3	emergency
W269	Orthopedic	3	emergency
W281	Orthopedic	3	
W283	Orthopedic	3	
W291	Orthopedic	3	
W293	Orthopedic	3	
W299	Orthopedic	3	
W301	Orthopedic	3	
W303	Orthopedic	3	
W304	Orthopedic	3	
W332	Orthopedic	4	emergency
W336	Orthopedic	3	
W361	Orthopedic	2	
W362	Orthopedic	2	
W371	Orthopedic	4	
W372	Orthopedic	4	
W373	Orthopedic	4	
W374	Orthopedic	4	
W378	Orthopedic	4	
W381	Orthopedic	4	
W382	Orthopedic	4	
W383	Orthopedic	4	
W384	Orthopedic	4	

W391	Orthopedic	4
W393	Orthopedic	4
W394	Orthopedic	4
W395	Orthopedic	4
W396	Orthopedic	4
W398	Orthopedic	4
W399	Orthopedic	4
W401	Orthopedic	4
W402	Orthopedic	4
W403	Orthopedic	4
W404	Orthopedic	4
W408	Orthopedic	4
W409	Orthopedic	4
W411	Orthopedic	4
W412	Orthopedic	4
W414	Orthopedic	4
W419	Orthopedic	4
W421	Orthopedic	4
W424	Orthopedic	4
W425	Orthopedic	4
W426	Orthopedic	4
W429	Orthopedic	4
W431	Orthopedic	4
W441	Orthopedic	4
W442	Orthopedic	4
W451	Orthopedic	4
W453	Orthopedic	4
W454	Orthopedic	4
W461	Orthopedic	4
W471	Orthopedic	4
W481	Orthopedic	4
W484	Orthopedic	4
W485	Orthopedic	4
W491	Orthopedic	3
W501	Orthopedic	3
W504	Orthopedic	3
W511	Orthopedic	3
W515	Orthopedic	3
W521	Orthopedic	3
W523	Orthopedic	3
W531	Orthopedic	3
W541	Orthopedic	3
W542	Orthopedic	3
W543	Orthopedic	3

W544	Orthopedic	3	
W551	Orthopedic	3	
W562	Orthopedic	3	
W571	Orthopedic	3	
W572	Orthopedic	3	
W581	Orthopedic	4	
W582	Orthopedic	4	
W589	Orthopedic	4	
W591	Orthopedic	2	
W593	Orthopedic	2	
W595	Orthopedic	2	
W596	Orthopedic	2	
W598	Orthopedic	2	
W601	Orthopedic	3	
W611	Orthopedic	3	
W621	Orthopedic	3	
W622	Orthopedic	3	
W629	Orthopedic	3	
W652	Orthopedic	4	emergency
W654	Orthopedic	4	emergency
W659	Orthopedic	4	emergency
W663	Orthopedic	4	emergency
W664	Orthopedic	4	emergency
W668	Orthopedic	4	emergency
W669	Orthopedic	4	emergency
W673	Orthopedic	4	emergency
W676	Orthopedic	4	emergency
W677	Orthopedic	4	emergency
W693	Orthopedic	4	
W702	Orthopedic	3	
W712	Orthopedic	3	
W742	Orthopedic	3	
W752	Orthopedic	3	
W758	Orthopedic	3	
W778	Orthopedic	3	
W779	Orthopedic	3	
W781	Orthopedic	3	
W788	Orthopedic	3	
W791	Orthopedic	3	
W792	Orthopedic	3	
W802	Orthopedic	3	
W803	Orthopedic	3	
W811	Orthopedic	3	
W818	Orthopedic	3	

W822	Orthopedic	2
W823	Orthopedic	2
W828	Orthopedic	2
W829	Orthopedic	2
W833	Orthopedic	2
W835	Orthopedic	2
W838	Orthopedic	2
W844	Orthopedic	2
W848	Orthopedic	2
W851	Orthopedic	2
W852	Orthopedic	2
W858	Orthopedic	2
W868	Orthopedic	2
W871	Orthopedic	2
W879	Orthopedic	2
W889	Orthopedic	2
W901	Orthopedic	2
W902	Orthopedic	2
W903	Orthopedic	2
W911	Orthopedic	2
W913	Orthopedic	2
W918	Orthopedic	2
W919	Orthopedic	2
W921	Orthopedic	2
W924	Orthopedic	2
W931	Orthopedic	4
W941	Orthopedic	4
W942	Orthopedic	4
W943	Orthopedic	4
W951	Orthopedic	4
W953	Orthopedic	4
W961	Orthopedic	4
W963	Orthopedic	4
W965	Orthopedic	4
W966	Orthopedic	4
W971	Orthopedic	4
W972	Orthopedic	4
W975	Orthopedic	4
W981	Orthopedic	4
W986	Orthopedic	4
X082	Orthopedic	3
X083	Orthopedic	3
X084	Orthopedic	3
X142	Orthopedic	5

X143	Orthopedic		5
X148	Orthopedic		5
X203	Orthopedic		3
S012	Plastic	2	
S049	Plastic	1	
S055	Plastic	1	
S065	Plastic	1	
S067	Plastic	1	
S068	Plastic	1	
S069	Plastic	1	
S131	Plastic	1	
S132	Plastic	1	
S142	Plastic	1	
S152	Plastic	1	
S159	Plastic	1	
S238	Plastic	3	
S249	Plastic	3	
S279	Plastic	3	
S314	Plastic	3	
S351	Plastic	3	
S352	Plastic	3	
S353	Plastic	3	
S358	Plastic	3	
S359	Plastic	3	
S379	Plastic	3	
S404	Plastic	3	
S411	Plastic	1	
S418	Plastic	1	
S421	Plastic	1	
S423	Plastic	1	
S424	Plastic	1	
S433	Plastic	1	
S454	Plastic	1	
S456	Plastic	1	
S471	Plastic	1	
S472	Plastic	1	
S474	Plastic	1	
S476	Plastic	1	
S504	Plastic	1	
S538	Plastic	1	
S551	Plastic	3	
S561	Plastic	3	
S563	Plastic	2	
S564	Plastic	1	

S569	Plastic	3
S571	Plastic	2
S573	Plastic	1
S574	Plastic	1
S575	Plastic	1
S578	Plastic	1
S582	Plastic	1
S604	Plastic	1
S608	Plastic	1
S641	Plastic	1
S662	Plastic	1
S681	Plastic	1
T521	Plastic	1
T525	Plastic	1
T529	Plastic	1
T541	Plastic	1
T561	Plastic	1
T591	Plastic	1
T592	Plastic	1
T598	Plastic	1
T962	Plastic	2
T968	Plastic	2
V011	Plastic	3
V082	Plastic	3
V092	Plastic	2
V143	Plastic	2
V144	Plastic	2
V152	Plastic	2
V198	Plastic	2
M011	Renal_tract_surgery	4
M013	Renal_tract_surgery	4
M015	Renal_tract_surgery	4
M019	Renal_tract_surgery	4
M021	Renal_tract_surgery	4
M022	Renal_tract_surgery	4
M025	Renal_tract_surgery	4
M029	Renal_tract_surgery	4
M039	Renal_tract_surgery	4
M051	Renal_tract_surgery	4
M062	Renal_tract_surgery	4
M064	Renal_tract_surgery	3
M093	Renal_tract_surgery	2
M094	Renal_tract_surgery	2
M119	Renal_tract_surgery	3

M131	Renal_tract_surgery	3
M132	Renal_tract_surgery	3
M135	Renal_tract_surgery	2
M136	Renal_tract_surgery	3
M137	Renal_tract_surgery	4
M138	Renal_tract_surgery	4
M141	Renal_tract_surgery	2
M164	Renal_tract_surgery	3
M165	Renal_tract_surgery	2
M181	Renal_tract_surgery	4
M193	Renal_tract_surgery	4
M195	Renal_tract_surgery	3
M198	Renal_tract_surgery	3
M212	Renal_tract_surgery	4
M253	Renal_tract_surgery	2
M271	Renal_tract_surgery	2
M272	Renal_tract_surgery	2
M273	Renal_tract_surgery	2
M274	Renal_tract_surgery	3
M275	Renal_tract_surgery	3
M278	Renal_tract_surgery	3
M281	Renal_tract_surgery	2
M282	Renal_tract_surgery	2
M283	Renal_tract_surgery	2
M288	Renal_tract_surgery	2
M292	Renal_tract_surgery	2
M293	Renal_tract_surgery	2
M295	Renal_tract_surgery	2
M298	Renal_tract_surgery	2
M301	Renal_tract_surgery	2
M302	Renal_tract_surgery	2
M309	Renal_tract_surgery	2
M311	Renal_tract_surgery	2
M318	Renal_tract_surgery	2
M326	Renal_tract_surgery	2
M332	Renal_tract_surgery	3
M334	Renal_tract_surgery	3
M335	Renal_tract_surgery	3
M341	Renal_tract_surgery	5
M342	Renal_tract_surgery	5
M343	Renal_tract_surgery	5
M348	Renal_tract_surgery	5
M351	Renal_tract_surgery	4
M375	Renal_tract_surgery	4

M378	Renal_tract_surgery	4
M382	Renal_tract_surgery	3
M391	Renal_tract_surgery	4
M418	Renal_tract_surgery	4
M421	Renal_tract_surgery	3
M422	Renal_tract_surgery	3
M423	Renal_tract_surgery	3
M432	Renal_tract_surgery	3
M441	Renal_tract_surgery	3
M442	Renal_tract_surgery	3
M443	Renal_tract_surgery	3
M444	Renal_tract_surgery	3
M448	Renal_tract_surgery	2
M451	Renal_tract_surgery	2
M452	Renal_tract_surgery	2
M453	Renal_tract_surgery	2
M455	Renal_tract_surgery	2
M459	Renal_tract_surgery	2
M471	Renal_tract_surgery	1
M472	Renal_tract_surgery	1
M473	Renal_tract_surgery	1
M478	Renal_tract_surgery	1
M479	Renal_tract_surgery	1
M492	Renal_tract_surgery	2
M493	Renal_tract_surgery	2
M494	Renal_tract_surgery	2
M495	Renal_tract_surgery	2
M496	Renal_tract_surgery	1
M498	Renal_tract_surgery	2
M611	Renal_tract_surgery	4
M612	Renal_tract_surgery	4
M614	Renal_tract_surgery	4
M618	Renal_tract_surgery	5
M619	Renal_tract_surgery	5
M642	Renal_tract_surgery	4
M647	Renal_tract_surgery	3
M648	Renal_tract_surgery	4
M651	Renal_tract_surgery	4
M653	Renal_tract_surgery	4
M654	Renal_tract_surgery	4
M655	Renal_tract_surgery	4
M658	Renal_tract_surgery	3
M659	Renal_tract_surgery	3
M662	Renal_tract_surgery	3

M668	Renal_tract_surgery	3
M672	Renal_tract_surgery	3
M676	Renal_tract_surgery	3
M678	Renal_tract_surgery	4
M702	Renal_tract_surgery	2
M703	Renal_tract_surgery	2
M706	Renal_tract_surgery	3
M708	Renal_tract_surgery	3
M711	Renal_tract_surgery	3
M712	Renal_tract_surgery	3
M722	Renal_tract_surgery	3
M736	Renal_tract_surgery	3
M761	Renal_tract_surgery	3
M763	Renal_tract_surgery	3
M764	Renal_tract_surgery	3
M766	Renal_tract_surgery	3
M792	Renal_tract_surgery	3
M793	Renal_tract_surgery	2
M814	Renal_tract_surgery	2
M851	Renal_tract_surgery	2
M868	Renal_tract_surgery	2
N012	Renal_tract_surgery	2
N051	Renal_tract_surgery	3
N063	Renal_tract_surgery	3
N068	Renal_tract_surgery	3
N071	Renal_tract_surgery	3
N111	Renal_tract_surgery	2
N113	Renal_tract_surgery	2
N114	Renal_tract_surgery	2
N118	Renal_tract_surgery	2
N132	Renal_tract_surgery	2
N153	Renal_tract_surgery	2
N171	Renal_tract_surgery	1
N201	Renal_tract_surgery	2
N262	Renal_tract_surgery	3
N271	Renal_tract_surgery	3
N282	Renal_tract_surgery	2
N283	Renal_tract_surgery	2
N289	Renal_tract_surgery	2
N291	Renal_tract_surgery	2
N292	Renal_tract_surgery	3
N303	Renal_tract_surgery	2
N304	Renal_tract_surgery	2
N325	Renal_tract_surgery	2

X451	Renal_tract_surgery	4	
A442	spinalcord_neurosurgery		5
A448	spinalcord_neurosurgery		5
A481	spinalcord_neurosurgery		5
A521	spinalcord_neurosurgery		5
A522	spinalcord_neurosurgery		3
A552	spinalcord_neurosurgery		3
V221	spine_neurosurgery	4	
V222	spine_neurosurgery	4	
V223	spine_neurosurgery	4	
V228	spine_neurosurgery	4	
V233	spine_neurosurgery	4	
V241	spine_neurosurgery	5	
V242	spine_neurosurgery	5	
V248	spine_neurosurgery	5	
V251	spine_neurosurgery	4	
V252	spine_neurosurgery	4	
V253	spine_neurosurgery	4	
V254	spine_neurosurgery	4	
V255	spine_neurosurgery	4	
V258	spine_neurosurgery	4	
V259	spine_neurosurgery	4	
V264	spine_neurosurgery	4	
V281	spine_neurosurgery	4	
V291	spine_neurosurgery	4	
V294	spine_neurosurgery	4	
V299	spine_neurosurgery	4	
V319	spine_neurosurgery	4	
V331	spine_neurosurgery	4	
V332	spine_neurosurgery	4	
V337	spine_neurosurgery	4	
V375	spine_neurosurgery	5	
V382	spine_neurosurgery	4	
V383	spine_neurosurgery	4	
V385	spine_neurosurgery	4	
V386	spine_neurosurgery	4	
V388	spine_neurosurgery	4	
V404	spine_neurosurgery	4	
V411	spine_neurosurgery	4	
V423	spine_neurosurgery	4	
V444	spine_neurosurgery	4	
V445	spine_neurosurgery	5	
V452	spine_neurosurgery	5	
V461	spine_neurosurgery	4	

V462	spine_neurosurgery		4
V468	spine_neurosurgery		4
V473	spine_neurosurgery		3
V499	spine_neurosurgery		4
V528	spine_neurosurgery		4
V544	spine_neurosurgery		2
V671	spine_neurosurgery		4
V672	spine_neurosurgery		4
E485	Thoracic	2	
E486	Thoracic	2	
E488	Thoracic	2	
E489	Thoracic	2	
E491	Thoracic	2	
E492	Thoracic	2	
E495	Thoracic	2	
E498	Thoracic	2	
E499	Thoracic	2	
E503	Thoracic	4	
E506	Thoracic	4	
E511	Thoracic	4	
E518	Thoracic	4	
E519	Thoracic	4	
E538	Thoracic	5	
E541	Thoracic	5	
E543	Thoracic	5	
E544	Thoracic	5	
E545	Thoracic	5	
E548	Thoracic	5	
E551	Thoracic	5	
E552	Thoracic	5	
E578	Thoracic	5	
E591	Thoracic	5	
E593	Thoracic	5	
E595	Thoracic	5	
E598	Thoracic	5	
E611	Thoracic	5	
E621	Thoracic	5	
E631	Thoracic	5	
E639	Thoracic	4	
T013	Thoracic	4	
T018	Thoracic	4	
T028	Thoracic	4	
T033	Thoracic	4	
T034	Thoracic	4	

T038	Thoracic	4
T039	Thoracic	4
T053	Thoracic	4
T054	Thoracic	3
T058	Thoracic	4
T071	Thoracic	5
T072	Thoracic	5
T088	Thoracic	5
T092	Thoracic	4
T094	Thoracic	5
T095	Thoracic	5
T102	Thoracic	5
T111	Thoracic	4
T112	Thoracic	4
T119	Thoracic	4
T121	Thoracic	4
T122	Thoracic	4
T123	Thoracic	4
T124	Thoracic	3
T129	Thoracic	3
T131	Thoracic	4
T141	Thoracic	3
X558	unspecified_surgery	
L393	Vascular_intervention	4
L394	Vascular_intervention	3
L431	Vascular_intervention	3
L434	Vascular_intervention	3
L472	Vascular_intervention	4
L473	Vascular_intervention	4
L541	Vascular_intervention	4
L544	Vascular_intervention	4
L631	Vascular_intervention	4
L632	Vascular_intervention	4
L633	Vascular_intervention	4
L634	Vascular_intervention	4
L635	Vascular_intervention	4
L665	Vascular_intervention	4
L667	Vascular_intervention	4
L671	Vascular_intervention	3
L713	Vascular_intervention	4
L718	Vascular_intervention	4
L721	Vascular_intervention	4
L726	Vascular_intervention	2
L728	Vascular_intervention	4

O011	Vascular_intervention	3	
O031	Vascular_intervention	3	
O041	Vascular_intervention	3	
L184	Vascular_surgery	5	
L194	Vascular_surgery	5	
L195	Vascular_surgery	5	
L196	Vascular_surgery	5	
L198	Vascular_surgery	5	
L199	Vascular_surgery	5	
L216	Vascular_surgery	5	
L236	Vascular_surgery	5	
L238	Vascular_surgery	5	
L271	Vascular_surgery	5	
L274	Vascular_surgery	5	
L276	Vascular_surgery	5	
L279	Vascular_surgery	5	
L281	Vascular_surgery	5	
L294	Vascular_surgery	5	
L295	Vascular_surgery	5	
L301	Vascular_surgery	5	
L302	Vascular_surgery	5	
L303	Vascular_surgery	5	
L312	Vascular_surgery	4	
L451	Vascular_surgery	5	
L514	Vascular_surgery	5	
L571	Vascular_surgery	5	
L572	Vascular_surgery	5	
L575	Vascular_surgery	5	
L579	Vascular_surgery	5	
L583	Vascular_surgery	5	emergency
L591	Vascular_surgery	5	
L592	Vascular_surgery	5	
L593	Vascular_surgery	5	
L595	Vascular_surgery	5	
L601	Vascular_surgery	5	
L602	Vascular_surgery	5	
L603	Vascular_surgery	5	
L608	Vascular_surgery	5	
L621	Vascular_surgery	5	
L622	Vascular_surgery	5	
L701	Vascular_surgery	5	
L703	Vascular_surgery	4	
L711	Vascular_surgery	5	
L742	Vascular_surgery	4	

L743	Vascular_surgery	4
L745	Vascular_surgery	4
L746	Vascular_surgery	4
L752	Vascular_surgery	4
L791	Vascular_surgery	4
L841	Vascular_surgery	2
L842	Vascular_surgery	2
L844	Vascular_surgery	2
L851	Vascular_surgery	2
L852	Vascular_surgery	2
L853	Vascular_surgery	2
L859	Vascular_surgery	2
L871	Vascular_surgery	2
L872	Vascular_surgery	2
L873	Vascular_surgery	2
L874	Vascular_surgery	2
L881	Vascular_surgery	2
L882	Vascular_surgery	2
L918	Vascular_surgery	2
L942	Vascular_surgery	2
L943	Vascular_surgery	2
L951	Vascular_surgery	2
L959	Vascular_surgery	2
L974	Vascular_surgery	2
X093	Vascular_Surgery	4
X095	Vascular_Surgery	4
X108	Vascular_Surgery	3
X111	Vascular_Surgery	3
X112	Vascular_Surgery	3
X118	Vascular_Surgery	3
X119	Vascular_Surgery	3
X121	Vascular_Surgery	4
X123	Vascular_Surgery	4
